# Effectiveness of Mobile-Delivered Exercise and Yoga Programs on Depressive Symptom Reduction in Employees: Randomized Controlled Trial

**DOI:** 10.2196/80287

**Published:** 2026-09-01

**Authors:** Jeong-Hyun Kim, So Young Yoo, Sohee Oh, Sun-Young Moon, Kyesan Lee, Ki-Hyeok Nam, Heyeon Park, Se Hee Jung, Hyo-Joon Choi, Hyo Jung Kim, Seok Im Lee, Namhee Kim, Myoungnam Song, Beomjun Min, Ji-Hye Lee, Jeong A Shin

**Affiliations:** 1 Department of Psychiatry, Seoul National University Bundang Hospital, 82 Gumi-ro 173 beon-gil, Bundang-gu, Seongnam-si, Gyeonggi-do, 13620, Republic of Korea, +82 31-787-2025, +82 31-787-4050; 2 Department of Public Health Medical Services, Seoul National University Bundang Hospital, Bundang, Republic of Korea; 3 Department of Psychiatry, College of Medicine, Seoul National University, Seoul, Republic of Korea; 4 Department of Psychiatry, Seoul Metropolitan Government-Seoul National University Boramae Medical Center, Seoul, Republic of Korea; 5 Interdisciplinary Program in Cognitive Science, Seoul National University, Seoul, Republic of Korea; 6 Medical Research Collaborating Center, Seoul Metropolitan Government Seoul National University Boramae Medical Center, Seoul, Republic of Korea; 7 Electronic Engineering, College of Electronics & Information, Kyunghee University, Yongin, Republic of Korea; 8 Korea Association of Sports Culture and Industry, Seoul, Republic of Korea; 9 Misoinfotech, Gwacheon, Republic of Korea; 10 Division of General Studies & Teaching Profession, Dongduk Women's University, Seoul, Republic of Korea; 11 Department of Rehabilitation Medicine, Seoul Metropolitan Government - Seoul National University Boramae Medical Center, Seoul, Republic of Korea; 12 Department of Rehabilitation Medicine, College of Medicine, Seoul National University, Seoul, Republic of Korea; 13 01arts Corporation, Seoul, Republic of Korea; 14 R&D Center, HYUNDAI MIB International Co., Ltd., Gunpo, Republic of Korea; 15 Seoul Chung Psychiatry Clinic, Seoul, Republic of Korea

**Keywords:** exercise, yoga, mental health, stress, depression, employee, mobile health, digital health

## Abstract

**Background:**

Mental health challenges such as stress and depression are prevalent among employees. Mobile health platforms that deliver exercise or yoga interventions offer a promising approach to improve mental health outcomes in this population.

**Objective:**

This study aimed to assess the effectiveness of 12-session adaptive moderate-intensity exercise and yoga programs delivered via a motion-detecting digital platform in reducing stress and depressive symptoms among employees.

**Methods:**

This was an unblinded, 3-arm, parallel-group, randomized controlled trial conducted at Seoul National University Bundang Hospital and Boramae Medical Center between November 2023 and January 2024. Eligible participants were full-time employees. Seventy-five participants were randomly assigned to an exercise, a yoga, or a cognitive behavioral therapy–based self-care control group using computer-generated randomization. The exercise and yoga groups engaged in motion-detecting, adaptive physical activity training, whereas the control group accessed mobile-based, self-directed stress management educational materials. The intervention was largely automated, with no individualized therapeutic guidance provided. Allocation was concealed until trial entry. All recruitment and outcome assessments were conducted in person at the hospitals. The primary outcomes were perceived stress and depressive symptoms, whereas the secondary outcomes included posttraumatic stress, insomnia severity, cognitive stress response, occupational stress, and burnout. Physiological outcomes were assessed using heart rate variability and electroencephalography. Measurements were collected at baseline, immediately after the intervention, and at 4-week follow-up. Data were analyzed using a multivariate linear model to evaluate the main effects of time, group, and time×group interactions.

**Results:**

Of the 75 randomized participants (exercise: n=24, 32%; yoga: n=25, 33.3%; and control: n=26, 34.7%), 71 (94.7%) who completed at least 9 of the 12 sessions (≥40 min each) were included in the outcome analysis (exercise: n=21, 29.5%; yoga: n=24, 33.8%; and control: n=26, 36.6%). For the coprimary outcomes, the group×time interaction for depressive symptoms (Patient Health Questionnaire-9) approached but did not reach the Bonferroni-corrected threshold (*F*_4,136_=2.71; *P*=.03; adjusted α=.025); however, planned pairwise comparisons revealed significantly greater improvement in the yoga group compared to the control group at 4-week follow-up (β=−3.67; adjusted *P*<.001). For the Perceived Stress Scale, the interaction was not significant (*P*=.29), although a significant main effect of time (*P*<.001) indicated overall stress reduction across all groups. For secondary outcomes, a significant group×time interaction was found for the Cognitive Stress Responses Scale (*P*=.003), indicating differential trajectories of improvement. The yoga group showed a consistent linear decrease, whereas the exercise group showed immediate but less sustained gains.

**Conclusions:**

Digitally delivered adaptive yoga programs demonstrated superior and sustained improvements in depressive symptoms and Cognitive Stress Responses Scale scores compared with the active cognitive behavioral therapy–based self-care control group. However, the exercise program showed more modest and less sustained effects, warranting further investigation using larger samples.

## Introduction

Mental health issues, including stress, depression, and anxiety, are major global health concerns, particularly among employees. Pressures in modern work environments, compounded by sedentary lifestyles, have led to an increased prevalence of psychological distress [1]. These mental health conditions not only affect individual well-being but also contribute to broader societal effects, including reduced productivity and increased health care costs [2]. Studies have shown that poor mental health among employees is associated with absenteeism, presenteeism, and lower workplace efficiency, highlighting the need for effective, accessible, and sustainable interventions [3]. Considering these challenges, there is growing interest in identifying effective, accessible, and sustainable interventions to improve mental health, particularly among the working population.

Physical exercise is associated with numerous mental health benefits, particularly in alleviating anxiety and depressive symptoms. Recent systematic reviews and meta-analyses have confirmed that physical activity can reduce psychological distress, improve mood, and enhance cognitive function [4,5]. These effects are believed to be mediated by various physiological mechanisms, including endorphin release, improved neuroplasticity, and regulation of the hypothalamic-pituitary-adrenal axis [5]. Despite these benefits, busy employees find adherence to regular exercise challenging, with time constraints and fatigue after long workdays being cited as common barriers [6].

Yoga has been shown to offer substantial mental health benefits as a mind-body intervention. Yoga integrates physical posture with breath control and mindfulness to provide a holistic approach to promoting physical and psychological well-being. Studies have suggested that yoga reduces stress and anxiety while enhancing mindfulness and relaxation [7]. Recent studies have demonstrated that yoga can significantly reduce symptoms of stress, anxiety, and depression while improving emotional regulation and social connectedness [8,9]. A 2024 randomized controlled trial (RCT) found that yoga can benefit mental health, with participants reporting improved self-compassion, extrinsic affect, and personal and communal spiritual well-being compared with control groups [10]. Moreover, yoga’s flexibility allows practitioners to choose options ranging from gentle meditative practices to more intense forms, thus aligning with their time constraints and physical capabilities. This makes yoga particularly well-suited to individuals with busy schedules, potentially addressing some of the challenges associated with adherence to more physically demanding exercise routines [11].

Although physical exercise and yoga have been validated as effective mental health interventions, they offer distinct benefits and challenges, particularly for busy employees. Yoga, owing to its low impact and flexible nature, may be more accessible and sustainable for individuals with limited time. In contrast, moderate-intensity exercise boosts vitality and energy, which may be particularly beneficial for individuals experiencing fatigue or low energy [12]. However, lack of motivation, busy work schedules, and family commitments can pose significant barriers to adherence [13].

Recently, the integration of digital tools, including mobile apps, into health care has expanded the scope of health interventions. Mobile health platforms are increasingly being used to deliver exercise and wellness programs, offering convenience and personalization [14]. Mobile technology can provide access to personalized training programs at the user’s convenience, overcoming traditional barriers to participation such as location, time, and access to expert instruction [15]. Wang et al [16] demonstrated that real-time feedback using smart wearable devices could improve postural control precision during balance training, suggesting that such systems enhance user engagement by providing immediate and actionable insights into movement accuracy.

This study evaluated the effects of a 12-session yoga program and an adaptive moderate-intensity physical exercise program using a mobile platform on employees’ mental health outcomes compared to those of an active control group based on cognitive behavioral therapy (CBT). The primary hypothesis was that yoga and physical exercise would positively influence mental health outcomes, such as reduced stress and depressive symptoms, compared to the control group. Furthermore, we hypothesized that these interventions induce beneficial changes in physiological markers, such as heart rate variability (HRV) and electroencephalography (EEG), and that differential effects may exist between the yoga and exercise groups on specific outcomes. By incorporating a mobile platform, we aimed to enhance the feasibility of the intervention by allowing participants to engage in programs at home and monitor their progress using real-time feedback.

## Methods

### Participants

The participants were enrolled via advertisements at Seoul National University Bundang Hospital and Boramae Medical Center between November 2023 and January 2024. The inclusion criteria were as follows: (1) employed adults aged 19 to 49 years; (2) individuals experiencing moderate-to-high levels of stress, defined as a Perceived Stress Scale (PSS) score of ≥14 at baseline; (3) individuals undergoing pharmacological treatment for psychiatric conditions, such as depression, anxiety disorders, or insomnia, who were eligible if their symptoms were stable and their medication dosages were not expected to change during the clinical trial; and (4) individuals who fully understood the clinical trial protocol and voluntarily consented to participate.

The exclusion criteria were as follows: (1) individuals aged <19 or >49 years; (2) those with dementia, intellectual disabilities, or other cognitive impairments; (3) individuals with seizure disorders, stroke, or other neurological conditions; (4) those with a history or current diagnosis of psychotic disorders, such as schizophrenia or bipolar disorder; (5) individuals with active suicidal ideation; (6) those using medications or having medical conditions (eg, heart or lung disease) that could affect the reliability of HRV testing; (7) individuals who received nonpharmacological psychiatric treatment or counseling within the past 6 months; and (8) individuals with physical limitations preventing them from participating in the exercise or yoga program.

The presence of psychiatric disorders was assessed using the Mini International Neuropsychiatric Interview, a short, structured psychiatric interview designed to identify a broad spectrum of psychiatric disorders as outlined in the *DSM-IV* (*Diagnostic and Statistical Manual of Mental Disorders* [Fourth Edition])*,* and the *ICD-10* (*International Statistical Classification of Diseases, Tenth Revision*). The Korean version of the Mini International Neuropsychiatric Interview has been shown to have good validity and reliability. The interviews, which typically took 15 to 30 minutes to complete, were conducted by 2 qualified psychologists with master’s degrees who were familiar with the tool.

The total sample size was calculated a priori using the G*Power software (version 3.1.9.4; Heinrich-Heine-Universität Düsseldorf). On the basis of previous meta-analyses of digital mental health interventions [17], we anticipated a large effect size (*f*=0.40). To achieve a statistical power of 0.80 with a significance level (α) of .05 for a 3-group ANOVA, a minimum of 66 participants (22 per group) was required. This provided a conservative power estimate for the multivariate linear model (MLM) used in the primary analysis. After accounting for a potential dropout rate of 10%, 75 participants were recruited.

### Measures and Assessments

#### Overview

The participants who provided consent underwent a baseline assessment. This assessment included demographic information, such as age, sex, marital status, education level, and occupation. It also included an evaluation of psychiatric diagnoses, medical history, and mental health–related questionnaires.

Changes in the PSS and Patient Health Questionnaire-9 (PHQ-9) scores for depressive symptoms were used as the primary psychological outcome measures. The secondary psychological outcomes included changes in the scores of scales assessing insomnia (Insomnia Severity Index), occupational stress (Korean Occupational Stress Scale [KOSS]), burnout (Maslach Burnout Inventory-General Survey), positive and negative affect (Positive and Negative Affect Schedule-Revised), posttraumatic stress symptoms (Posttraumatic Stress Disorder Checklist-5 [PCL-5]), interoceptive awareness (Multidimensional Assessment of Interoceptive Awareness [K-MAIA]), mindfulness (Five Facet Mindfulness Questionnaire [FFMQ]), and cognitive stress responses (Cognitive Stress Response Scale [CSRS]).

Originally developed by Cohen et al [18], the PSS is a 14-item scale; however, a modified 10-item version is commonly used [19,20]. This scale measures the degree to which individuals have perceived their lives as unpredictable, uncontrollable, or overwhelming in the past month. Higher scores indicate greater perceived stress. Total scores range from 0 to 40, with each item rated on a 5-point Likert scale, ranging from 0 (never) to 4 (very often).

PHQ-9 is a self-report depression screening tool that assesses the severity of depressive symptoms [21]. It comprises items based on 9 diagnostic criteria for major depressive disorder from DSM-IV. Total scores range from 0 to 27, with higher scores indicating more severe depressive symptoms.

Insomnia Severity Index was developed by Morin [22] to assess insomnia severity based on the diagnostic criteria of *DSM-IV* and the International Classification of Sleep Disorders. It comprises 7 items that examine the type and severity of insomnia over the past 2 weeks, satisfaction with sleep, daytime functioning, impact of sleep disturbances, and distress caused by sleep difficulties. Total scores range from 0 to 28, with higher scores indicating more severe insomnia.

The KOSS-Short Form is a shortened version of the KOSS consisting of 19 items reduced from the original 43. Each item is rated on a 4-point Likert scale, ranging from 1 (not at all) to 4 (very much). Higher scores indicate greater job stress experienced in the past month. The scale comprises 8 domains: physical environment, job demands, lack of job control, insufficient social support, job insecurity, organizational injustice, inappropriate rewards, and work-life imbalance. It was designed for easy field application [23].

The Maslach Burnout Inventory-General Survey, developed by Schaufeli et al [24] and adapted by Shin [25], was used to measure participants’ current levels of job burnout. This scale assesses 3 core aspects of workplace burnout: exhaustion, cynicism, and professional efficacy. Each item is rated on a 7-point Likert scale, ranging from 0 (strongly disagree) to 6 (strongly agree). Higher scores on the exhaustion and cynicism domains and lower scores on the professional efficacy domain indicate higher degrees of job burnout.

To measure the participants’ positive and negative affect, we used the Positive and Negative Affect Schedule developed by Watson et al [26] and adapted by Park and Lee [27]. It assesses the emotions experienced over the past week using 20 items, with participants rating their affective experiences on a 5-point Likert scale.

We used the PCL-5 to measure the severity of posttraumatic stress disorder (PTSD) symptoms. The original PCL was developed by Weathers et al [28] and revised by Weathers et al [29] to align with the *DSM-5* (*Diagnostic and Statistical Manual of Mental Disorders* [Fifth Edition]) diagnostic criteria. The Korean version of the PCL-5 was validated by Lee et al [30]. The PCL-5 assesses the severity of symptoms experienced in the past month in relation to traumatic events. It comprises 20 items rated on a 5-point Likert scale, ranging from 0 (not at all) to 4 (extremely). Higher scores indicate more severe PTSD symptoms. PCL-5 includes 4 subscales: intrusion, avoidance, negative alterations in cognition and mood, and hyperarousal.

To assess participants’ habitual body awareness, we used the K-MAIA, which was validated by Gim et al [31] based on the original scale developed by Mehling et al [32]. K-MAIA consists of 32 items rated on a 7-point Likert scale, ranging from 0 (not at all) to 6 (always). It comprises 6 subscales: noticing, accepting, attention regulation, mind-body connection awareness, returning to the body, and trusting. Noticing assesses awareness of positive, negative, and neutral bodily sensations by measuring sensitivity to physical sensations. Accepting evaluates the tendency to neither avoid nor divert attention from physical discomfort or the ability to not experience emotional distress from such discomfort. Attention regulation refers to the ability to control and maintain focus on bodily sensations. Mind-body connection awareness measures recognition of the connection between bodily sensations and emotions. Returning to the body refers to the ability to manage distress through attention to bodily sensations, leading to internal insight. Trusting reflects the extent to which one’s body is experienced as safe and trustworthy.

To measure participants’ habitual mindfulness levels, we used the Korean version of the FFMQ (K-FFMQ) validated by Won and Kim [33] and based on the original scale developed by Baer et al [34]. K-FFMQ includes 39 items rated on a 7-point Likert scale, ranging from 1 (not at all) to 7 (always). Mindfulness is defined as observing the flow of internal and external stimuli as they occur without judgment and is considered an adaptive response that helps prevent automatic, impulsive, and maladaptive behaviors [34]. This scale comprises 5 factors. Nonreactivity refers to the ability to refrain from immediate reactions to internal experiences without becoming overwhelmed. Observing means attending to and noticing internal phenomena (eg, bodily sensations, thoughts, and emotions) and external stimuli (eg, sounds and smells). Description measures the ability to label and describe observed experiences in words. Acting with awareness refers to fully engaging in the present activity without distraction. Nonjudgment refers to refraining from making evaluations such as good or bad, right or wrong, or valuable or worthless.

To assess participants’ cognitive stress responses, we used the CSRS developed by Koh and Park [35]. This scale evaluates 3 dimensions of the cognitive stress response: extreme negative, aggressive-hostile, and self-depreciative thoughts. It comprises 21 items rated on a 5-point Likert scale, ranging from 0 (not at all) to 4 (very much). The test-retest reliability ranges from 0.87 to 0.95, and the internal consistency is 0.94.

**Figure 1.**
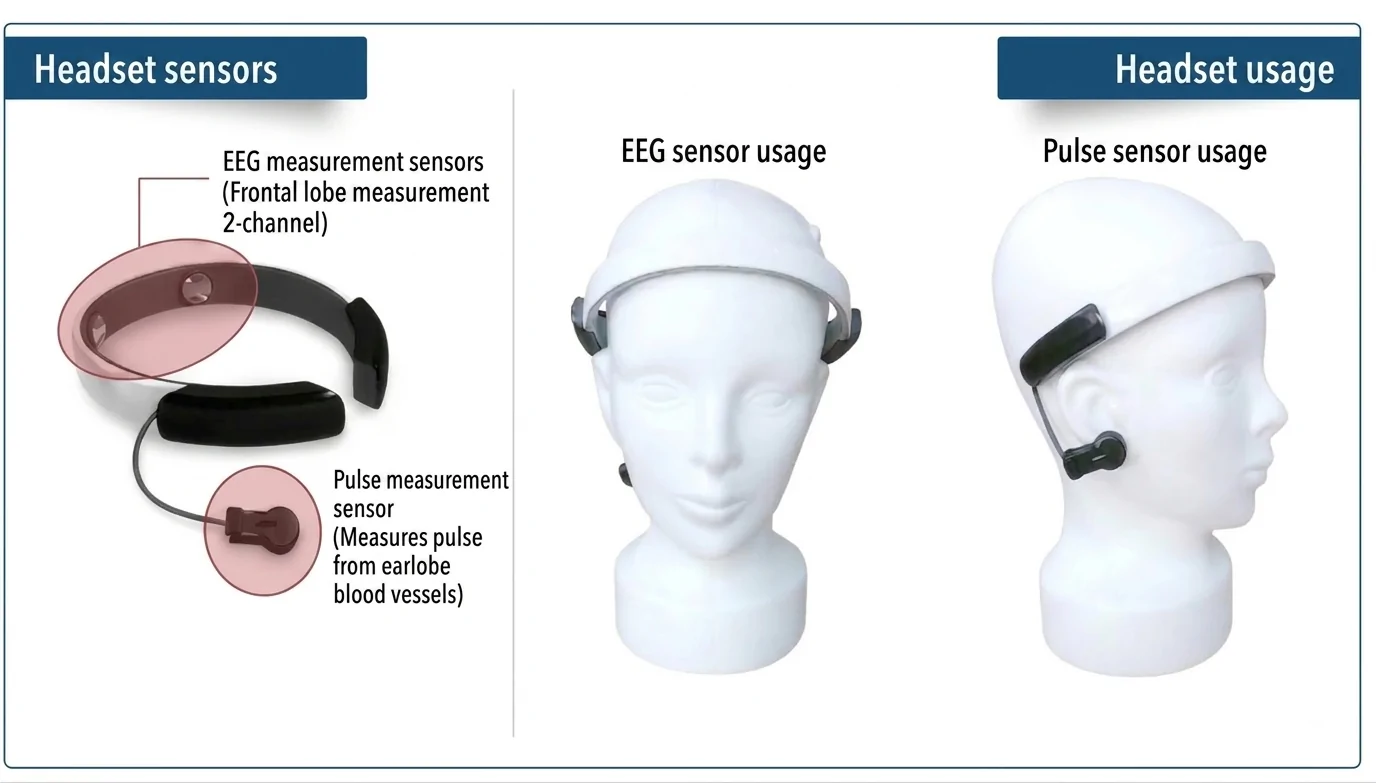
neuroNicle FX2 device. EEG: electroencephalography.

In addition, we measured physiological variables, including HRV and EEG, which are widely used as stress-related physiological biosignals [35-37]. EEG and HRV were measured using the neuroNicle FX2 (OMNI C&S, Inc) [38]. The device was equipped with 2 EEG monopolar electrodes positioned over the left and right prefrontal areas (FP1 and FP2, respectively), along with ground and reference electrodes attached to a clamp on the right earlobe (Figure 1). Additionally, one pulse wave sensor (photoplethysmograph [PPG]) was attached to the same clamp to measure HRV. EEG and PPG data were simultaneously recorded. The device was approved by the South Korean Ministry of Food and Drug Safety (authorization 16‐4837). EEG and HRV measurements were recorded over a 5-minute period in a resting state with eyes closed, and the results were analyzed.

**Figure 2.**
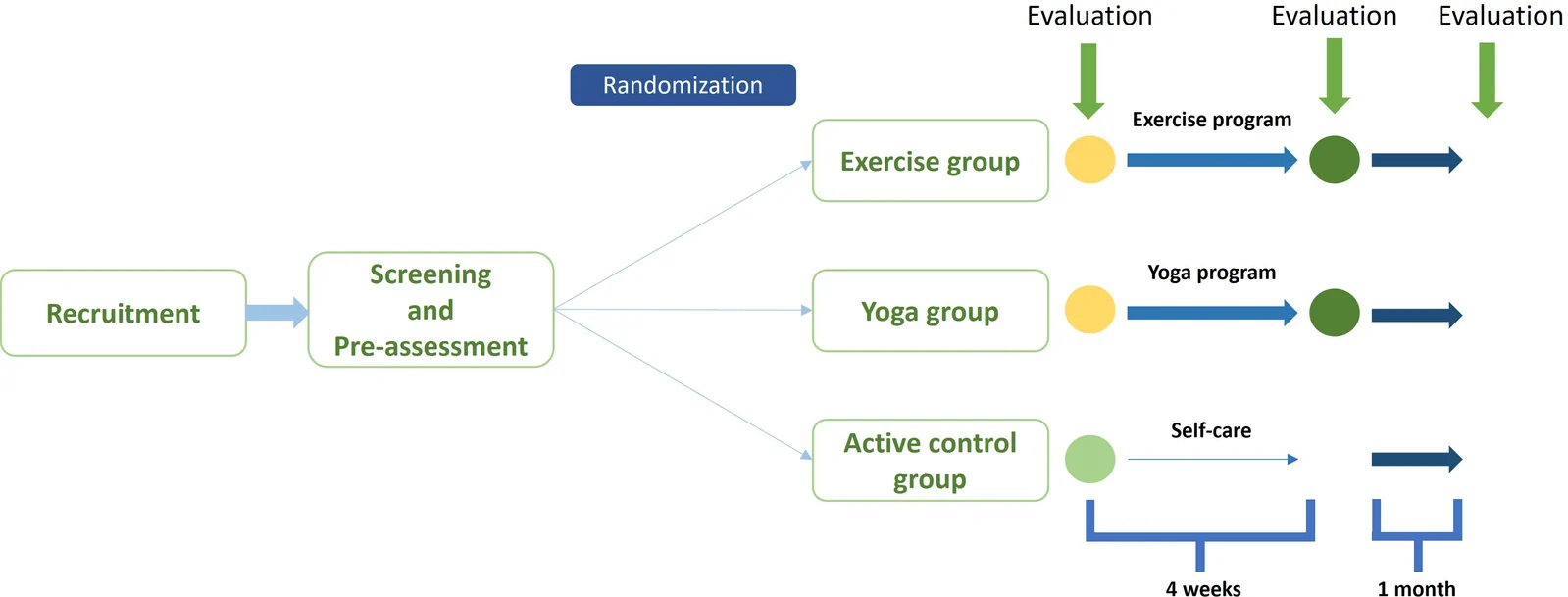
Assessment process of the study.

Throughout the study, all self-reported psychological, cognitive, and physiological indices were measured at 3 time points: during enrollment (baseline assessment), immediately after training (post-training assessment), and 4 weeks after training (1-mo follow-up assessment; Figure 2). Although psychological outcome measures (eg, PSS, PHQ-9, and CSRS) are typically validated for self-report, their administration in this study was conducted in person at the hospital during assessment visits, rather than purely online or remotely. This approach was used to ensure data quality and minimize potential technical issues or self-report bias often associated with unproctored web-based questionnaires. In addition, participants completed a structured user satisfaction and usability questionnaire, comprising four 4-point Likert-scale items and 6 closed-ended items (3 single-response and 3 multiple-response items in which up to 2 options could be selected), at their final visit (Multimedia Appendix 1).

#### Apparatus

The reliability of the prefrontal EEG marker from the neuroNicle FX2 device [38] was previously established by Choi et al [39], who reported no significant differences in the means of the tested variables between the prefrontal and occipital regions.

The raw data collected by the equipment were processed using TeleScan (OMNI C&S, Inc). Ocular, muscular, and other types of artifacts were detected using an automatic removal algorithm, and contaminated periods longer than 10 seconds were removed. For EEG, the band-pass filter (infinite impulse response Butterworth filters, high-pass filter: first order with fc=2.6 Hz; low-pass filter: eighth order with fc=43 Hz), with the 3 to 43 Hz range, was applied to the data. All data were digitized in continuous recording mode (5 min of recording, 250-Hz sampling rate, and 15-bit resolution). For HRV, the PPG waveform and heartbeat time interval of 4 ms for each heartbeat were detected using the pulse wave sensor. Raw EEG and PPG data were transmitted to the host device in real time. In the host device, a fast Fourier transform was applied to calculate the power spectrum of the EEG and HRV data.

The power spectra of the θ (4‐8 Hz), α (8‐12 Hz), low β (12‐15 Hz), β (15‐20 Hz), high β (20‐30 Hz), and γ (30‐40 Hz) frequency bands were calculated from the EEG data. In addition, relaxation (ratio of α to high β power) and concentration (ratio of low β to θ power) indices were calculated from the EEG data. For HRV parameters, the power of the low-frequency (0.04‐0.15 Hz) and high-frequency (0.15‐0.4 Hz) bands was calculated. All variables were normalized according to sex and age.

### Platform of Fitness and Yoga

A fitness platform was developed to enable participants to engage in exercise or yoga programs. To improve motion recognition accuracy, usability testing was conducted in 3 phases: the first phase involved office workers, the second included medical professionals, and the third targeted the general public in home settings. The evaluation focused on key performance indicators, including user interface intuitiveness, functional accessibility, system stability, and latency. The tests identified specific areas for improvement, such as menu complexity, ambiguity in instructional text, and suboptimal user inteUser Interface intuitiveness. Recognizing that these issues could increase user-entry barriers and adversely affect adoption rates, corrective refinements were implemented prior to this clinical trial. Furthermore, the testing phases revealed that environmental variables, such as background interference and camera positioning, affected motion recognition accuracy. Consequently, the recognition algorithm was further optimized, and a comprehensive user manual was developed to ensure consistent performance across diverse environments. To ensure accuracy and quality of information, this study used Redmine, an open-source project management and defect-tracking platform, to oversee the development lifecycle. White box testing methodologies were used for logical defect detection and structural verification. We used a suite of automated testing tools, such as unit, Pytest, and SonarQube, to ensure the precision of the software’s internal logic. The version of the platform evaluated was the initial release (version 1.0, “frozen” during the trial period). No major content or functional changes, bug fixes, or downtimes occurred that might have influenced the study results. Institutional affiliations were not displayed on the digital intervention platforms and were disclosed only during the consent process.

### Randomization and Intervention Conditions

#### Overview

This study used a parallel-group randomized controlled design. Participants were assigned to 3 groups by 1:1:1 block randomization (stratified by organization and sex) with randomly selected block sizes (3, 6, or 6) using REDCap (Vanderbilt University) tools hosted at the Medical Research Collaborating Center of Seoul National University Bundang Hospital. REDCap generates randomization codes with SAS (SAS Institute). Owing to the nature of the intervention, participants and intervention providers were not blinded; however, the allocation sequence was concealed from participants until they entered the trial and from the investigators until the end of the study. Participants were aware of the 3 intervention types but were not informed about the intervention of interest or the comparators.

Participants accessed the platform via mobile devices or online and followed the instructed movements displayed on the screen (Multimedia Appendix 2). Participants’ joint movements were recorded and analyzed in real time during the sessions. They were also provided feedback on their exercise performance during relaxation and transitions between movements to ensure and enhance their participation and engagement. Only participants’ skeletal movement data (body movement) were collected to check the accuracy of their movements, without facial recognition or screen recording. Participants’ movements were tracked, and they were alerted with pop-up notifications of any deviations from the prescribed exercise or yoga movements, encouraging adherence. Motivational pop-up messages were displayed at the end of each session. Participants were instructed to access a designated internet address via a web browser using their unique ID to engage in the exercise, yoga, or CBT-based self-care program specifically developed for this study. During the study period, participants were advised to refrain from engaging in any exercise beyond the prescribed program. The program was conducted in a quiet and comfortable location of the participant’s choosing. To minimize environmental influences, participants were instructed to use the same location for each session, ensuring that it was a space where they could engage in exercise or yoga without interruption. Notably, participants did not need to visit the hospital to complete the exercise or yoga sessions.

The control group participants who practiced CBT-based self-care were instructed to access a designated website through a web browser to view educational materials based on CBT. This paper covers the definition of stress, its signs and symptoms, and strategies for its management.

Participants accessed the intervention platforms free of charge using their own devices and internet connections and received 300,000 KRW (US $231) after completing the 4-week study. The intervention itself (exercise and yoga sessions) was fully automated, relying on motion detection technology to provide real-time objective feedback. Human involvement during the trial was limited to recruitment, in-person baseline or outcome assessments at the hospital, and basic technical support, as needed. This minimal human involvement was designed to reflect the potential for low-cost routine applications (see section *Discussion*). The use of the interventions was defined and measured by the completion of prescribed 12 sessions within the 4-week study period. This adherence metric was monitored using the platform’s backend log file analysis. The ultimate measure of usage intensity had a high completion rate (71/75 randomized participants). During the intervention period and follow-up, no significant harmful or unintended effects were reported in any of the groups (exercise, yoga, and control).

#### Exercise Program

The exercise program was designed by the Korea Association of Sports Culture and Industry, an expert exercise organization involved in this study. On the basis of a literature review, the program was designed to alleviate the symptoms of conditions, such as depression, anxiety, and posttraumatic stress, by considering the intensity, frequency, duration, and type of exercise. These exercises included adaptive moderate-intensity exercises. Each session comprised warm-up, training, and cool-down phases. The program was conducted over 12 sessions, with each session lasting 50 minutes, held 3 times a week for 4 weeks.

- Warm-up (8 min): Stretching, which focuses on flexibility and balance, was incorporated to enhance joint mobility before the main exercise. This phase, which included self-stretching exercises, lasted for 8 minutes.
- Training (26 min): The main exercises focused on core strength and balance, with strength exercises being conducted for 26 minutes.
- Cool down (16 min): Designed to calm the sympathetic nervous system, this 16-minute phase included balance stabilization, static stretching, breathing exercises, mindset focus, and relaxation movements.

The protocol also incorporated expert recommendations from a researcher in the Rehabilitation Medicine Department at the Boramae Medical Center, ensuring a comprehensive approach to the exercise regimen. Multimedia Appendices 3 and 4 present the detailed protocols for the exercise program.

#### Yoga Program

An example of the movements in the program protocol is presented in Multimedia Appendix 5, and the schedule of the 4-week yoga program is presented in Multimedia Appendix 6.

#### CBT-Based Self-Care Program

The CBT-based active control group received educational materials aimed at stress management delivered via the platform through either web or mobile access. This occurred 3 times per week over 4 weeks, for a total of 12 sessions. The materials were based on CBT principles, with a focus on applying the content learned in the “Let’s Take a Moment” section to actionable behavior. This group served as an active control intervention to control for the nonspecific effects of attention, time, and digital platform access. The program was designed to encourage CBT-based self-care practices appropriate for an active control group. Multimedia Appendix 7 presents the protocol for the CBT-based stress management educational materials.

The CBT-based self-care control group was advised to refrain from exercising during the study period.

Adherence was monitored using the platform’s backend log file analysis. A minimum completion threshold of 9 of 12 sessions (≥75%), each lasting at least 40 minutes, was required for inclusion in the outcome analysis. The mean number of completed sessions did not differ significantly between groups. The quality of the exercise and yoga performance was ensured through the platform’s real-time motion detection system, which provided automated corrective feedback when movement deviations were detected.

### Statistical Analysis

Demographic variables and baseline psychological measurements were analyzed using ANOVA or Kruskal-Wallis tests for continuous variables, whereas chi-square or Fisher exact tests were used for categorical variables to compare the 3 groups.

The primary analysis followed the intention-to-treat principle using an MLM for repeated measures [40,41], accommodating potential missing data points. This model evaluated the effects of group, time, and their interaction across 3 time points: baseline, post-training (4 wk), and follow-up (8 wk). A first-order autoregressive (AR(1)) correlation structure was specified to account for within-subject correlations, and baseline PSS scores were included as covariates to minimize confounding effects.

To control for the family-wise error rate (FWER), a hierarchical testing strategy was adopted for the 2 co-primary outcomes (PSS and PHQ-9). First, the group×time interaction was evaluated at a Bonferroni-corrected significance level of 0.025 (0.05/2 outcomes). Regardless of the interaction significance, prespecified planned pairwise comparisons (yoga vs control, exercise vs control, and yoga vs exercise) were performed at each post-baseline time point using a Bonferroni-adjusted alpha of 0.0083 (0.025 per 3 groups) to test our a priori hypotheses. For secondary outcomes, although treated as exploratory, a Bonferroni-adjusted alpha of 0.0167 (0.05 per 3 groups) was applied for pairwise comparisons to maintain statistical stringency.

In accordance with CONSORT (Consolidated Standards of Reporting Trials) reporting standards, results of group comparisons were reported as mean differences (β), adjusted 95% CIs, and adjusted *P* values. These CIs were calculated to align with corrected α levels (.0083 and .0167, respectively) to ensure a transparent interpretation of both statistical significance and clinical magnitude. The overall model parameters (eg, *F*, df_1_, and df_2_) are reported in the table.

Frontal θ power was defined as the primary physiological outcome of interest. All other EEG frequency bands (α, β, and γ) were treated as secondary or exploratory outcomes. Consequently, no formal adjustment for multiple testing was applied to these exploratory measures, and the results should be interpreted as hypothesis generating.

For the user satisfaction and usability questionnaire administered at the final visit (Multimedia Appendix 1), the four 4-point Likert-scale items were compared between the 2 active intervention groups (exercise vs yoga) using 2-tailed independent samples *t* tests, with Cohen *d* as the effect size because these items referred specifically to the assigned exercise or yoga program. The 6 closed-ended items—3 single-response items and 3 multiple-response items in which participants could select up to 2 options—were summarized as frequencies and percentages, and between-group differences in the endorsement of each response option were examined using Fisher exact tests. These analyses were exploratory and were not adjusted for multiple comparisons.

All statistical analyses were performed using SAS (version 9.4).

### Ethical Considerations

The study protocol for this RCT was approved by the institutional review boards of Seoul National University Bundang Hospital (approval B-2309-855-301) and Boramae Medical Center in South Korea (approval 30-2023-106), and the trial was registered at ClinicalTrials.gov (NCT06620783). At their initial onsite visit, all participants were provided with comprehensive written and verbal information regarding the study purpose, procedures, and interventions and subsequently provided written informed consent in person prior to participation. Participants were informed of their right to withdraw from the study at any time without penalty, and the original consent also covered secondary analyses without the need for additional consent. All data collected, including self-reported psychological, cognitive, and physiological indices, were handled with strict confidentiality; data were deidentified and stored using unique research IDs. Participants received compensation of KRW 100,000 (US $77) for their time and travel expenses at each of the 3 required evaluation visits (baseline, post-intervention, and 4-week follow-up), amounting to a maximum total compensation of KRW 300,000 (US $231).

During the intervention period and follow-up, no significant harmful or unintended effects were reported in any of the groups (exercise, yoga, and control).

## Results

### Overview

**Figure 3.**
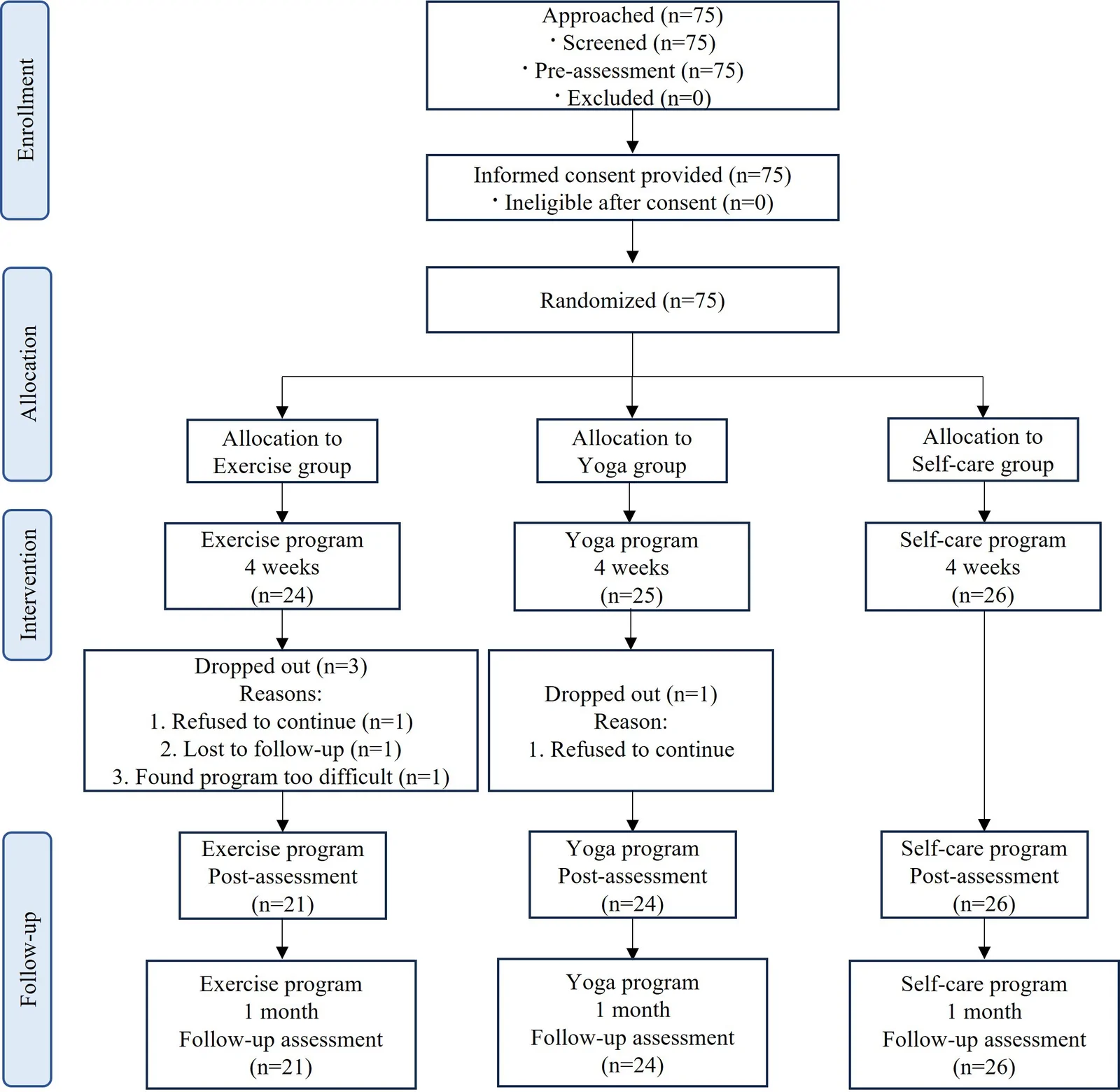
A flow diagram of the study process.

Of the 75 randomized participants (exercise, 24/75, 32%; yoga, 25/75, 33.3%; and control, 26/75, 34.7%), 71 who completed at least nine of the 12 sessions, with a minimum duration of 40 minutes per session, were included in the outcome analysis (exercise, 21/71, 29.6%; yoga, 24/71, 33.8%; and control, 26/71, 36.6%). Figure 3 presents a flow diagram of participant enrollment and retention. Specific reasons for attrition included personal time constraints (2/75, 2.67%), exercise program difficulty (1/75, 1.33%), and inability to establish follow-up contact (1/75, 1.33%). Participants’ average age was 34.14 (SD 6.18) years, and 77.3% (58/75) were women. No significant group differences were observed in demographic characteristics, such as age, sex, marital status, or education level at baseline (all *P*>.05; Table 1).

Both the exercise and yoga interventions led to improved mental health outcomes over time. The yoga group demonstrated the most pronounced and sustained benefits, while the exercise group showed immediate gains in some cognitive measures.

**Table 1.** Demographic and clinical characteristics of each group at baseline.

Variables	Exercise (n=24)	Yoga (n=25)	CBT^a^-based self-care (n=26)	*P* value
Age (years), mean (SD)	34.58 (6.49)	34.60 (6.10)	34.04 (6.19)	.94
Sex (female), n (%)	18 (75)	20 (80)	20 (76.92)	.91
Marital status (married), n (%)	7 (29.17)	12 (48)	8 (30.77)	.31
Education level, n (%)				.66
Bachelor’s degree or higher	19 (79.17)	22 (88)	23 (88.46)	
Job, n (%)				.69
Nurse	3 (12.50)	8 (32)	6 (23.08)	
Researcher	10 (41.67)	10 (40)	12 (46.15)	
Office worker	8 (33.33)	4 (16)	5 (19.23)	
Other	3 (12.50)	3 (12)	3 (11.54)	
PSS^b^_total, mean (SD)	21.42 (3.74)	21.88 (4.02)	19.27 (3.55)	.03
PHQ^c^_total, mean (SD)	6.63 (3.89)	8.08 (5.59)	6.88 (3.80)	.72
ISI^d^_total, mean (SD)	9.67 (5.40)	9.24 (4.87)	9.23 (4.99)	.97
KOSS-SF^e^_total, mean (SD)	42.75 (8.24)	43.36 (8.08)	43.04 (7.22)	.99
MBI-GS^f^_total, mean (SD)	47.83 (9.25)	49.60 (8.09)	48.15 (6.86)	.66
PANAS-R^g^_total, mean (SD)	43.92 (10.06)	47.04 (11.29)	45 (9.30)	.87
PCL-5^h^_total, mean (SD)	14.13 (16.16)	13.96 (14.82)	9.27 (12.94)	.31
Intrusion	3.79 (4.87)	3.44 (3.59)	2.35 (3.59)	.28
Avoidance	2.13 (2.52)	2.16 (2.03)	1.46 (2.14)	.32
Alterations in cognition and mood	4.92 (6.13)	4.92 (6.37)	3.46 (5.21)	.60
Hyperarousal	3.29 (3.78)	3.64 (5.06)	2.00 (3.25)	.39
K-MAIA^i^_total, mean (SD)	91.25 (31.30)	97.92 (31.95)	105.12 (20.11)	.20
K-FFMQ^j^_total, mean (SD)	160.79 (28.54)	163.88 (21.35)	164.46 (23.32)	.44
CSRS^k^_total, mean (SD)	39.29 (28.54)	35.68 (11.99)	32.96 (9.25)	.44
Extreme negative thoughts	6.46 (2.36)	6.44 (2.77)	5.81 (2.08)	.53
Aggressive hostile thoughts	5.38 (2.46)	4.84 (1.11)	4.73 (1.66)	.33
Self-depreciative thoughts	13.79 (6.88)	11.72 (5.65)	10.73 (3.95)	.26

a CBT: cognitive behavioral therapy.

b PSS: Perceived Stress Scale.

c PHQ: Patient Health Questionnaire-9.

d ISI: Insomnia Severity Index.

e KOSS-SF: Korean Occupational Stress Scale-Short Form.

f MBI-GS: Maslach Burnout Inventory-General Survey.

g PANAS-R: Positive and Negative Affect Schedule-Revised.

h PCL-5: Posttraumatic Stress Disorder Checklist-5.

i K-MAIA: Korean version of the Multidimensional Assessment of Interoceptive Awareness.

j K-FFMQ: Korean version of Five Facet Mindfulness Questionnaire.

k CSRS: Cognitive Stress Response Scale.

### Primary Outcomes

**Figure 4.**
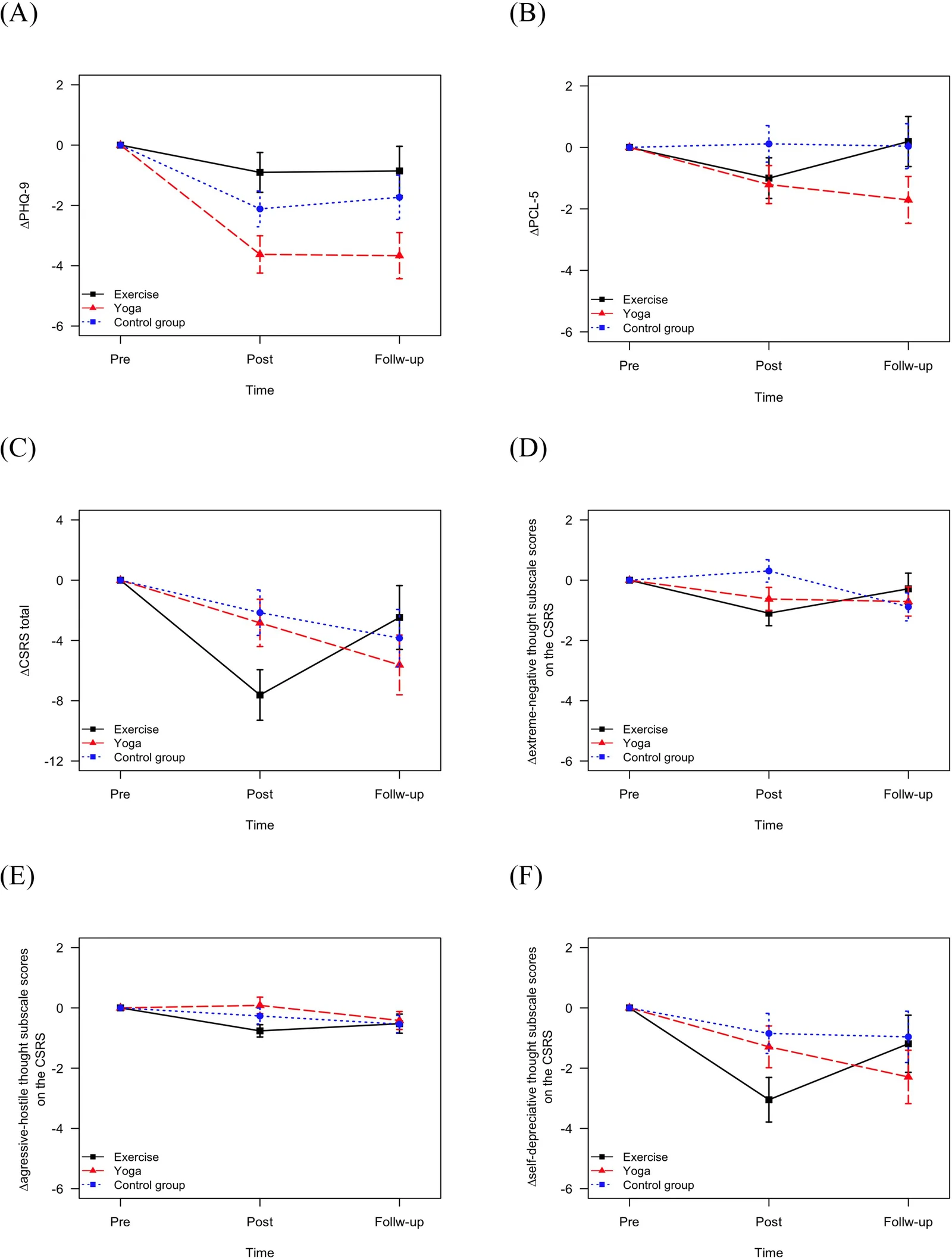
Changes in scores of Patient Health Questionnaire (PHQ)-9, Posttraumatic Stress Disorder Checklist-5, and Cognitive Stress Response Scale (CSRS).

The group×time interaction for PHQ-9 did not reach the prespecified Bonferroni-corrected significance level of 0.025 (*F*_4,136_=2.71; *P*=.03). However, planned pairwise comparisons revealed a significant reduction in depressive symptoms for the yoga group compared to the control group at the 4-week follow-up (β=−3.67, 95% CI −5.64 to −1.69; adjusted *P*<.001). No significant differences were observed between the exercise and control groups at any time point (Figure 4A).

We also analyzed changes in another primary outcome: the PSS. The MLM showed that the group×time interaction was not statistically significant (*F*_4,136_=1.27; *P*=.29), indicating that the trajectory of stress reduction did not differ significantly between the yoga, exercise, and control groups. However, a significant main effect of time was observed (*F*_2,136_=38.56; *P*<.001), confirming a general reduction in perceived stress across all participants from baseline to the 4-week follow-up. The main effect of group was not significant (*F*_2,68_=1.13; *P*=.33). The results, including the *F* statistics and *P* values, are summarized in Table 2, while Table 3 presents between-group differences and within-group changes in items from baseline (adjusted 95% CI).

**Table 2.** Results of the multivariate linear model for repeated measures.

Outcome and effect	*F* statistics (*df*)	*P* value	Time	Least squares mean (SE)		
				Exercise	Yoga	Self-care
PSS^a^						
Group	1.13 (2, 68)	.33	Baseline	21.76 (0.77)	21.96 (0.82)	19.27 (0.68)
Time	38.56 (2, 136)	<.001	4W^b^	17.24 (0.89)	16.83 (1.20)	15.96 (0.83)
Group×time	1.27 (4, 136)	.29	8W^c^	16.71 (1.31)	16.50 (1.24)	16.35 (0.77)
PHQ^d^ total						
Group	2.49 (2, 67)	.09	Baseline	6.02 (0.63)	7.66 (0.77)	7.87 (0.71)
Time	20.13 (2, 136)	<.001	4W	5.11 (0.56)	4.04 (0.46)	5.76 (0.59)
Group×time	2.71 (4, 136)	.03	8W	5.16 (0.75)	4 (0.47)	6.14 (0.53)
Hyperarousal (PCL-5^e^ subscale)						
Group	0.24 (2, 67)	.79	Baseline	2.64 (0.65)	3.35 (0.81)	2.70 (0.60)
Time	1.53 (2, 136)	.22	4W	1.64 (0.51)	2.14 (0.48)	2.81 (0.53)
Group×time	2.61 (4, 136)	.04	8W	2.83 (0.76)	1.64 (0.48)	2.73 (0.56)
Extreme negative thought						
Group	0.17 (2, 67)	.85	Baseline	6.26 (0.39)	6.23 (0.46)	6.24 (0.43)
Time	3.77 (2, 136)	.03	4W	5.16 (0.35)	5.60 (0.42)	6.54 (0.43)
Group×time	4.04 (4, 136)	.004	8W	5.97 (0.42)	5.52 (0.35)	5.35 (0.42)
Aggressive hostile thought						
Group	0.26 (2, 67)	.77	Baseline	4.95 (0.27)	4.76 (0.20)	4.92 (0.32)
Time	7.44 (2, 136)	<.001	4W	4.19 (0.13)	4.84 (0.29)	4.65 (0.18)
Group×time	3.05 (4, 136)	.02	8W	4.43 (0.11)	4.34 (0.17)	4.38 (0.12)
Self-depreciative thought						
Group	1.09 (2, 67)	.34	Baseline	13.17 (1.18)	11.33 (0.95)	11.60 (0.72)
Time	38.56 (2, 136)	<.001	4W	10.13 (0.66)	10.03 (0.78)	10.75 (0.47)
Group×time	3.03 (4, 136)	.02	8W	11.98 (1.00)	9.03 (0.50)	10.64 (0.62)
CSRS^f^ total						
Group	0.71 (2, 67)	.495	Baseline	37.27 (2.44)	34.44 (1.77)	35.49 (1.48)
Time	10.99 (2, 136)	<.001	4W	29.65 (1.62)	31.60 (1.72)	33.34 (1.23)
Group×time	4.28 (4, 136)	.003	8W	34.79 (2.27)	28.81 (1.09)	31.65 (1.36)

a PSS: Perceived Stress Scale.

b 4W: 4 weeks.

c 8W: 8 weeks.

d PHQ: Patient Health Questionnaire-9.

e PCL-5: Posttraumatic Stress Disorder Checklist-5.

f CSRS: Cognitive Stress Response Scale.

**Table 3.** Between-group changes and within-group differences in items from baseline^a^.

Outcome, comparison, and time	Exercise	*P* value	Yoga	*P* value	Self-care	*P* value
PSS^b^						
Mean (SD)						
4W^c^	−4.52 (4.98)	<.001	−5.13 (4.44)	<.001	−3.31 (3.26)	<.001
8W^d^	−5.05 (6.55)	.002	−5.46 (5.06)	<.001	−2.92 (4.17)	.002
Between-group difference (95% CI)						
4W	−1.22 (−4.51 to 2.08)	.33	−1.82 (−4.73 to 1.09)	.10	Reference	—^e^
8W	−2.12 (−6.43 to 2.18)	.19	−2.54 (−5.99 to 0.92)	.05	Reference	—
Within-group change (95% CI)						
4W	−4.52 (−7.36 to −1.69)	<.001	−5.13 (−7.50 to −2.75)	<.001	−3.31 (−4.99 to −1.63)	<.001
8W	−5.05 (−8.78 to −1.31)	<.001	−5.46 (−8.16 to −2.75)	<.001	−2.92 (−5.07 to −0.78)	<.001
PHQ^f^ total score						
Mean (SD)						
4W	−0.90 (2.77)	.15	−3.63 (3.57)	<.001	−2.12 (3.17)	.002
8W	−0.86 (2.74)	.17	−3.67 (3.69)	<.001	−1.73 (3.46)	.02
Between-group difference (95% CI)						
4W	1.21 (−1.06 to 3.48)	.16	−1.51 (−4.02 to 1.00)	.11	Reference	—
8W	0.87 (−1.50 to 3.25)	.33	−1.94 (−4.60 to 0.72)	.05	Reference	—
Within-group change (95% CI)						
4W	−0.90 (−2.49 to 0.68)	.13	−3.63 (−5.54 to −1.71)	<.001	−2.12 (−3.75 to −0.48)	<.001
8W	−0.86 (−2.42 to 0.71)	.14	−3.67 (−5.64 to −1.69)	<.001	−1.73 (−3.51 to 0.05)	.01
Hyperarousal (PCL-5^g^)						
Mean (SD)						
4W	−1.00 (2.98)	.14	−1.21 (4.15)	.17	0.12 (3.06)	.85
8W	0.19 (2.98)	.77	−1.71 (3.39)	.02	0.04 (3.00)	.95
Between-group difference (95% CI)						
4W	−1.12 (−3.22 to 0.98)	.20	−1.32 (−3.79 to 1.14)	.20	Reference	—
8W	0.15 (−1.93 to 2.23)	.86	−1.75 (−3.90 to 0.41)	.05	Reference	—
Within-group change (95% CI)						
4W	−1.00 (−2.54 to 0.54)	.12	−1.21 (−3.22 to 0.80)	.15	0.12 (−1.31 to 1.54)	.85
8W	0.19 (−1.35 to 1.73)	.76	−1.71 (−3.35 to −0.06)^h^	.01	0.04 (−1.36 to 1.44)	.95
Extreme negative thought						
Mean (SD)						
4W	−1.10 (2.00)	.02	−0.63 (2.22)	.18	0.31 (1.72)	.37
8W	−0.29 (1.74)	.46	−0.71 (2.39)	.16	−0.88 (1.80)	.02
Between-group difference (95% CI)						
4W	−1.40 (−2.71 to −0.10)^h^	.01	−0.93 (−2.27 to 0.41)	.09	Reference	—
8W	0.60 (−0.63 to 1.83)	.24	0.18 (−1.25 to 1.60)	.77	Reference	—
Within-group change (95% CI)						
4W	−1.10 (−2.13 to −0.06)^h^	.01	−0.63 (−1.70 to 0.45)	.16	0.31 (−0.49 to 1.11)	.35
8W	−0.29 (−1.18 to 0.61)	.44	−0.71 (−1.86 to 0.45)	.14	−0.88 (−1.72 to −0.05)^h^	.01
Aggressive hostile Thought						
Mean (SD)						
4W	−0.76 (1.48)	.03	0.08 (1.41)	.78	−0.27 (1.89)	.47
8W	−0.52 (1.29)	.08	−0.42 (0.97)	.05	−0.54 (1.33)	.05
Between-group difference (95% CI)						
4W	−0.49 (−1.66 to 0.67)	.31	0.35 (−0.76 to 1.47)	.44	Reference	—
8W	0.01 (−0.90 to 0.93)	.97	0.12 (−0.66 to 0.90)	.71	Reference	—
Within-group change (95% CI)						
4W	−0.76 (−1.53 to 0.00)	.02	0.08 (−0.60 to 0.77)	.77	−0.27 (−1.15 to 0.61)	.46
8W	−0.52 (−1.19 to 0.14)	.06	−0.42 (−0.89 to 0.06)	.03	−0.54 (−1.16 to 0.08)	.04

a Statistical significance: *P* values and 95% CIs reflect alpha levels adjusted for multiple comparisons (Bonferroni correction) and baseline PSS imbalances, as specified in *Methods*.

b PSS: Perceived Stress Scale.

c 4W: 4 weeks.

d 8W: 8 weeks.

e Not applicable.

f PHQ: Patient Health Questionnaire-9.

g PCL-5: Posttraumatic Stress Disorder Checklist-5.

h *P*<adjusted α (primary outcomes: .008; secondary outcomes: .017).

### Secondary Outcomes

#### Overview

Similarly, a significant time×group interaction effect (*F*_4,136_=2.61; *P*=.04) was observed for hyperarousal symptoms, as measured by the PCL-5 subscale (Figure 4). Although pairwise comparisons did not show significant between-group differences at specific time points, a trend analysis indicated that the yoga group exhibited a significant linear decrease in hyperarousal symptoms over time, with an average reduction of 0.854 points per month (95% CI 0.18-1.52; *P*=.01). The exercise group showed a nonsignificant downward trend, whereas the control group showed no significant change. These findings suggest that the yoga intervention led to a continuous decrease in hyperarousal symptoms during the study period.

Cognitive stress responses, as measured by the CSRS total scores, showed a significant time×group interaction effect (*F*_4,136_=4.28; *P*=.003; Figure 4). At the post-training assessment, the exercise group showed the most substantial reduction in total CSRS scores; however, this effect diminished at the 1-month follow-up. In contrast, both yoga and control groups demonstrated significant linear decreases in CSRS scores over time. Notably, the yoga group exhibited a more pronounced average reduction of 2.81 points per month (95% CI 1.27-4.36; *P*<.001), while the control group showed an average reduction of 1.923 points per month (95% CI 0.54-3.30; *P*=.007). The exercise group showed significant improvements immediately post-training (post vs pre: β=−7.62, 95% CI −13.18 to −2.06; *P*_adj_<.01); however, some of this gain was lost by follow-up. Conversely, the yoga and control groups showed a continuous and significant decrease in CSRS scores during the study period.

As a subscale of CSRS, extreme negative thoughts showed a significant time×group interaction effect (*F*_4,136_=4.04; *P*=.004; Figure 4), indicating that changes in extreme negative thoughts over time differed across groups. The exercise group showed a substantial reduction in extreme negative thoughts post-training; however, this effect was not sustained at 1-month follow-up. In contrast, the control group demonstrated a significant decrease between the post-training and follow-up assessments, with an average reduction of 1.192 points (95% CI 0.42-1.97; *P*=.003). The yoga group showed no significant changes over time. These findings suggest that the exercise intervention led to an immediate decrease in extreme negative thoughts, although this effect diminished over time. Conversely, the control group experienced a delayed yet significant reduction, whereas the yoga group did not exhibit notable changes on this subscale.

Aggressive hostile thoughts also showed a significant time×group interaction effect (*F*_4,136_=3.05; *P*=.02; Figure 4). The exercise group showed the greatest reduction in aggressive hostile thoughts post-training. The control group showed a decreasing trend over time (β=.269, 95% CI 0.02-0.52; *P*=.04). However, the yoga group showed a decreased effect after the post-training point. The self-depreciative thought subscale of the CSRS also had a significant time×group interaction effect (*F*_4,136_=3.03; *P*=.02), with the yoga group showing the greatest sustained reduction (β=1.146, 95% CI 0.50- 1.79; *P*=.006; Figure 4). The exercise group showed a significant change immediately post-training, which was reversed after the training intervention ended.

**Figure 5.**
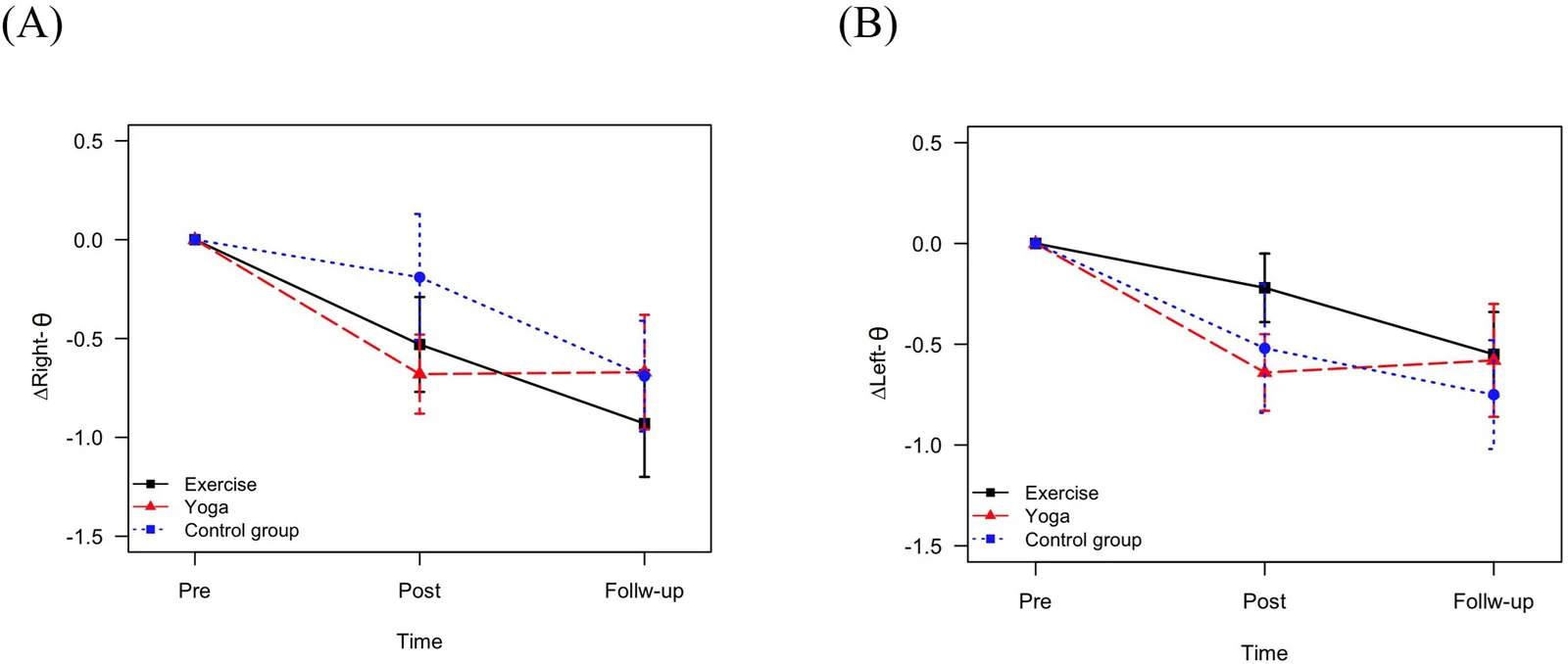
Changes in the power of the θ frequency band of (A) the right and (B) the left electroencephalogram channel at 3 time points.

Regarding biological markers, significant time effects were observed for both right and left EEG θ power (right θ: *F*_2,135_=12.06; *P*<.001; and left θ: *F*_2,135_=10.37; *P*<.001). As shown in Figure 5A, for right θ power, post-training levels were significantly lower than baseline levels (β=.45, 95% CI 0.15-0.76; *P*=.004), with a further decrease observed at the 1-month follow-up compared to baseline (β=.753, 95% CI 0.43-1.07; *P*<.001). However, the difference between post-training and follow-up θ power was not statistically significant (β=.302, 95% CI −0.04 to 0.65; *P*=.09). A comparable pattern was observed for the left θ power, as depicted in Figure 5. Post-training levels significantly decreased compared to baseline (β=.47, 95% CI 0.19-0.75; *P*=.001). This reduction remained significant at follow-up (β=.631, 95% CI 0.33-0.93; *P*<.001). No significant differences were found between the post-training and follow-up measurements (β=.16, 95% CI −0.15 to 0.48; *P*=.31). Other frequency bands, including α, β, and γ, did not show significant changes between pre- and post-training assessments. Furthermore, no significant group effects or group×time interactions were observed for any frequency band, including θ. Expanding on previous findings, no statistically significant changes were observed in the main HRV indices across time points.

#### User Satisfaction and Usability

Responses to the user satisfaction and usability questionnaire are summarized in Multimedia Appendix 1. For the 4 Likert-scale items, the yoga group rated their assigned program as significantly more helpful than the exercise group for stress relief (mean 2.83, SD 0.70 vs mean 2.33, SD 0.66 on a 4-point scale; *t*_43_=−2.45; *P*=.02; Cohen *d*=0.73) and for mental health management (mean 2.88, SD 0.61 vs mean 2.48, SD 0.60; *t*_43_=−2.20; *P*=.03; Cohen *d*=0.66). Perceived helpfulness of the platform for overall health management (*P*=.10) and ease of use of the platform (*P*=.47) did not differ significantly between the 2 groups.

For the 6 closed-ended items, response distributions were largely comparable between the exercise and yoga groups, and no between-group differences reached significance for desired improvements (Q5), perceived inconveniences (Q6), prior and intended platform use (Q7), main reasons for using the program (Q8), or main barriers to engagement (Q10). The only significant difference was for preferred platform functions (Q9), where participants in the exercise group more frequently endorsed “referring to a variety of exercise programs” than those in the yoga group (57.1% vs 25.0%; Fisher exact *P*=.04). Descriptively, the most frequently endorsed inconvenience in both groups was a preference for compatibility across multiple devices (76.2% and 75.0% in the exercise and yoga groups, respectively), and the most frequently endorsed barrier to sustained engagement was insufficient personal motivation to continue (45.5% and 62.5%, respectively). Full item-level frequencies, percentages, and test statistics are provided in Multimedia Appendix 1.

## Discussion

### Principal Findings

This study aimed to assess whether a 12-session mobile-delivered exercise and yoga programs could effectively reduce stress and depressive symptoms among employees. An RCT demonstrated that both an adaptive moderate-intensity exercise program and a yoga program delivered via a digital platform significantly improved mental health outcomes among employees with moderate-to-high stress compared with a CBT-based self-care control condition. Both interventions decreased depressive symptoms, hyperarousal, and maladaptive CSRS scores. In particular, the yoga intervention showed the most pronounced and sustained benefits across a range of psychological measures. The significant group×time interactions for depressive symptoms, hyperarousal, and CSRS indicated that the trajectories of improvement differed by group, with the yoga group often showing the greatest improvement over time. This is consistent with research on the mental health benefits of mindfulness-based practices, such as yoga, which have been shown to alleviate anxiety and depressive symptoms [10]. A significant main effect of time was observed on frontal θ power; however, no significant group or group×time interaction effects were found. The exercise group also showed improvements in most outcomes, although in some cases, these were not as large or sustained as those in the yoga group. The yoga group demonstrated the greatest and most sustained improvements, whereas the exercise group showed immediate but less sustained gains in secondary cognitive measures. These results suggest differential efficacy patterns among the intervention modalities.

Furthermore, CSRS scores, particularly for extreme negative, aggressive hostile, and self-depreciative thoughts, decreased more prominently in the yoga and exercise groups than in the control group. These findings suggest that physical activity and mind-body interventions can effectively reduce cognitive distortions associated with stress. Such decreases in negative thought patterns may have contributed to the overall improvement in mental health observed in this study.

In addition to psychological outcomes, this study explored brainwave activity, focusing specifically on θ power in the prefrontal cortex, which is commonly associated with cognitive processes, such as attention and memory. We observed a significant decrease in θ power after both interventions across all groups. Elevated θ power in resting states has been associated with poor cognitive functioning [42], including deficits in attention and executive function. The overall reduction in resting frontal θ power over time may be consistent with changes in neural markers associated with stress and arousal regulation. However, given the absence of cognitive performance measures, this interpretation is exploratory and should be viewed with caution [43]. This interpretation is supported by the results of previous studies. Thornberry et al [43] demonstrated that decreases in θ power are associated with improved spatial learning. Brzezicka et al [44] reported that reduced θ power in the dorsolateral prefrontal cortex, especially under a high cognitive load, predicts better task performance, which allows the brain to manage cognitive demands more effectively. The overall reduction in θ power observed across all groups may reflect improved arousal and attention regulation, possibly enhanced by the mindfulness components inherent in the yoga sessions. However, the absence of a significant group×time interaction precludes attributing this effect specifically to yoga.

### Comparison With Previous Studies

Our results are consistent with those of a growing body of literature highlighting the mental health benefits of exercise and yoga interventions. A recent systematic review by Singh et al [45] found that physical activity interventions can significantly reduce depression, anxiety, and psychological distress in various populations. Similarly, our exercise intervention reduced depressive symptoms and stress-related outcomes. The observed improvements in the exercise group align with the findings of Arida and Teixeira-Machado [46], who noted that exercise contributes to neuroplasticity and cognitive reserve, which are crucial for brain resilience to cognitive impairment.

Our findings on cognitive resilience and stress responses complement those of Shields et al [47], who found that collegiate athletes exhibited cognitive resilience through adaptations in mood and stress responses during a competitive season, suggesting that regular physical activity supports mental health and cognitive flexibility. Furthermore, Brush et al [48] showed that 8 weeks of moderate-intensity aerobic exercise can reduce depressive symptoms and influence neural indicators of reward and cognitive control in young adults with major depression, indicating potential neurobiological pathways for exercise effects and paralleling our EEG findings.

Bouthillier and Asmundson [49], Tseng et al [50], and Soteriades et al [51] demonstrated the efficacy of physical exercise (including aerobic and resistance training) in reducing anxiety, PTSD symptoms, and occupational stress in patients with anxiety disorders, middle-aged adults with poor sleep, and firefighters, respectively. By including physiological stress measures (HRV and EEG), our study provides additional evidence that such interventions can induce favorable changes in stress-related biomarkers, although our HRV results did not reach significance, possibly because of the relatively short intervention period.

Previous studies conducted on trauma-exposed and high-stress populations have supported the benefits of combining mind-body practices with exercise. Goldstein et al [52] reported that an integrative exercise program for veterans with PTSD reduced symptoms, whereas Rosenbaum et al [53] observed that an exercise program tailored to police officers with PTSD improved psychological outcomes. Similarly, our yoga program, which included mindfulness and relaxation components, produced notable improvements in hyperarousal and negative thought patterns, reflecting outcomes particularly relevant to stress and trauma-related conditions. McKeon et al [54] also showed that a mental health–informed physical activity intervention delivered via social media was feasible for first responders, suggesting that digital delivery can be effective, a finding echoed by the high adherence and engagement observed via our digital platform.

Our results on physical activity and stress align with epidemiological findings such as those of LeardMann et al [55], who observed an association between regular vigorous exercise and a lower incidence of PTSD symptoms in military personnel. In our study, participants in the exercise and yoga groups engaged in structured physical activity and showed corresponding decreases in stress and hyperarousal, supporting the protective and ameliorative effects of exercise on stress-related outcomes. Schuch et al [4] and Mikkelsen et al [5] found that physical exercise reduces anxiety and depression, likely through endorphin release and enhanced neuroplasticity. The decreases in depressive symptoms and improvements in hypervigilance and cognitive responses observed in our study align with these physiological mechanisms.

Our findings emphasize the importance of exercise intensity and enjoyment. Paolucci et al [56] noted that both high-intensity interval training and moderate continuous training could reduce depression and inflammation, although intensity matters and high intensity may increase perceived stress. In the exercise program, we chose moderate-intensity exercise and observed mental health benefits without evidence of increased stress, suggesting that a tolerable intensity level is crucial for psychological benefits. Similarly, the yoga program, which was generally low-to-moderate intensity, yielded strong benefits, consistent with the idea that the optimal intensity for mental health improvements might not necessarily be maximal.

The general improvements in mental health observed in this study are supported by large-scale data, such as those of Chekroud et al [57], who found that individuals who exercise have a significantly lower mental health burden. In that study, all exercise types were associated with better mental health, with decreases in mental health burden ranging from 12% to 22% compared to nonexercisers. We observed robust improvements in the exercise and yoga groups, reinforcing the idea that regular physical activity, whether traditional exercise or yoga, can substantially improve mental well-being. Notably, Chekroud et al [57] also found that certain group-based exercises and mindfulness practices (eg, yoga and tai chi) had larger effects than some other forms of activity, a trend mirrored in our study in which the yoga group often experienced equal or greater benefits than the conventional exercise group.

Our study adds to existing evidence on the efficacy of yoga in occupational contexts. Rhodes et al [58] demonstrated the benefits of yoga in women with treatment-resistant PTSD, and Shohani et al [59] demonstrated its positive effects on stress, anxiety, and depression in women. Jeter et al [60] found that a yoga program for police trainees improved mood and stress, whereas Weathers-Meyer et al [61] reported that firefighters who participated in an 8-week yoga program experienced reduced PTSD symptoms, negative affect, and trait anxiety. Similarly, our yoga program improved mood and stress (depressive symptoms and CSRS). Fang and Li [62] found that nurses who regularly practiced yoga reported better sleep quality and lower work stress, highlighting the broad benefits of yoga.

The comparable effectiveness of the CBT-based self-care control and yoga intervention groups may reflect the active psychological mechanisms embedded in both interventions. Self-guided CBT and psychoeducation, even in brief, have significantly reduced depressive symptoms in occupational populations [63]. This suggests that the control group did not serve as a passive comparator but instead offered evidence-based psychological tools with known antidepressant efficacy. Furthermore, stress regulation and mindfulness-based practices in yoga may overlap with CBT techniques through shared mechanisms of emotion regulation, attentional control, and cognitive reappraisal [64]. These parallels could account for the similar symptom reduction observed in both groups.

In contrast, the relatively weaker performance of the exercise group may be attributable to several pragmatic and methodological factors. First, the intervention was delivered remotely, without live supervision. Prior meta-analyses suggest that structured and professionally supervised exercise interventions tend to be associated with greater improvements in depressive symptoms compared to structured formats [65]. Second, real-world barriers, such as fatigue after long workdays and time constraints, may adversely affect engagement with exercise sessions [66]. Finally, the 4-week intervention duration may not have been sufficient to produce robust antidepressant effects from exercise [67], whereas the cognitive and emotional benefits of CBT-based self-care or yoga may manifest more quickly.

The user satisfaction findings converged with the primary outcome results. The yoga group, which showed the most pronounced and sustained improvements in depressive symptoms and cognitive stress responses, also rated their program as significantly more helpful for both stress relief and mental health management than the exercise group. This concordance between subjective perceptions of benefit and objective changes in psychological outcomes supports the internal consistency of our findings and suggests that the superior efficacy of the yoga intervention was accompanied by a correspondingly favorable user experience. The usability feedback also carries practical implications for future platform development. Across both groups, the most frequently endorsed request was for compatibility across multiple devices, and the most frequently reported barrier to sustained engagement was insufficient personal motivation to continue. These observations indicate that broadening device support and incorporating motivation-enhancing features (eg, progress reports, reminders, and social or gamified elements) may further improve adherence and engagement when such digitally delivered programs are scaled to routine workplace settings.

### Suggestions and Limitations

These findings have important clinical implications. First, yoga is a viable and accessible intervention, and exercise may complement it in reducing workplace stress and improving employee mental health. Given that these programs were delivered via a mobile platform, our study supports the potential of digital health solutions to reach employees with time or accessibility constraints. Employers and health professionals should consider incorporating such programs into workplace wellness initiatives to address stress and mental health proactively.

Second, the distinct advantages of yoga suggest that mind-body exercises may be particularly useful for individuals who struggle with stress-related cognitive distortions or heightened arousal. However, the exercise program showed meaningful improvements in vitality and energy, consistent with findings indicating that exercise boosts energy levels. A combined approach that offers both traditional exercises and yoga can cater to personal preferences and target a broader range of outcomes.

Despite these promising results, this study had several limitations. First, the relatively short intervention period (12 sessions over 4 wk) may limit the generalizability of the findings. Although this study demonstrated short-term improvements in mental health outcomes, it remains unclear whether these benefits can be sustained in the long term. Future studies should include longer follow-up periods to assess the durability of the observed effects. Second, our sample size (N=75, three groups) was calculated to detect large effects (*f*=0.40). A larger sample size would increase statistical power. Third, the study relied on self-reported measures of psychological outcomes, which could be subject to response bias. Objective measures of stress, such as cortisol levels, were not obtained in this study but could strengthen future research. Fourth, although we attempted to control for participant behavior outside the interventions (eg, advising against extra exercise), we cannot fully rule out external factors influencing the outcomes (eg, changes in work stress or personal life events during the study). Fifth, the control group received a CBT-based self-care program that, while intended to be minimal, might have provided some benefits (eg, stress management education). This may have diluted between-group differences. Nevertheless, the inclusion of basic psychoeducation in the control condition made our findings more applicable to real-world settings in which doing nothing is often unethical or impractical. More importantly, by comparing the interventions to active digital control, the study successfully isolated the differential impact of physical and mind-body components (yoga and exercise) from the nonspecific effects of general engagement and digital access. The observed significant group×time interactions validate that the specific mechanisms of the active interventions produced differential trajectories of mental health improvement compared to CBT-based self-care control. Sixth, the homogeneity of the sample—predominantly female, young, urban, and well-educated employees—may limit the applicability of the findings to other populations, such as men, older adults, rural workers, and those with lower digital literacy. The Korean cultural context may also influence intervention uptake. Seventh, the stringent exclusion criteria ensured participant safety but may limit applicability to employees with comorbid psychiatric or physical conditions. Eighth, several elements of this RCT may differ from routine application. In-person assessments and follow-ups conducted at the hospital provided a level of accountability and reduced loss to follow-up, which might not exist in purely remote routine applications. Additionally, the use of unique IDs and concealed allocations, when necessary, would be streamlined in a real-world scenario. The omission of in-person assessments in a routine setting might affect retention rates, underscoring the need for future studies on effective remote engagement strategies (eg, gamification or peer support). Ninth, EEG analyses were exploratory, and we did not apply formal corrections for multiple comparisons across frequency bands. As such, the observed time-related reductions in θ power should be considered hypothesis generating and require replication in future studies with preregistered EEG outcomes and appropriate controls for multiple testing. Finally, although the study successfully used a digital platform to deliver interventions, technical issues or user proficiency may have influenced engagement and outcomes. In addition, the analyses of the user satisfaction and usability questionnaire were exploratory and were not corrected for multiple comparisons; given the small subgroup sizes, these results should be interpreted with caution and regarded as hypothesis generating.

Despite these limitations, this study highlighted several unmet needs and areas for future research. First, further investigations are required to explore the long-term effects of digital mental health interventions, particularly in diverse workplaces. Understanding how different populations engage with digital platforms and whether the benefits of yoga and exercise persist beyond the initial intervention period is crucial for upscaling these programs.

Future research should focus on refining the digital delivery of mental health interventions. For example, incorporating gamification or social support elements may motivate users to adhere to the programs. Finally, studies comparing these interventions across different psychological distress levels are required. Although this study primarily focused on individuals with moderate-to-high stress, future research should investigate whether these findings hold true for individuals with lower baseline stress or severe mental health conditions. This could help tailor interventions for specific mental health needs, ensuring that the most appropriate treatment is provided.

### Conclusions

This study provides evidence that digitally delivered adaptive yoga programs demonstrate superior and sustained improvements in depressive symptoms and CSRS compared to an active CBT-based self-care control. However, the exercise program showed more modest and less sustained effects, which warrant further investigation using larger samples. The use of a motion-detecting digital platform demonstrated high feasibility and engagement, suggesting that mobile health interventions can overcome common barriers to exercise (eg, time and accessibility) in employee populations. Our findings support the integration of regular physical activity, including mind-body practices such as yoga, into workplace wellness programs to promote mental well-being. Large-scale studies with longer follow-up periods are warranted to confirm these results, examine the long-term maintenance of benefits, and evaluate the cost-effectiveness of such interventions. Employers and health care providers should consider leveraging digital health platforms to deliver structured mind-body programs, particularly yoga, to reduce workplace stress and enhance overall mental health among employees; however, the role of exercise warrants further investigation.

## Supplementary material

10.2196/80287 Multimedia Appendix 1 User satisfaction and usability questionnaire items and item-level results.

10.2196/80287 Multimedia Appendix 2 The screenshots of the exercise, yoga, and control platform.

10.2196/80287 Multimedia Appendix 3 Schedule for the 4-week adaptive moderate-intensity exercise program.

10.2196/80287 Multimedia Appendix 4 Examples of movements included in the exercise program protocol.

10.2196/80287 Multimedia Appendix 5 Example of movements in the yoga program.

10.2196/80287 Multimedia Appendix 6 Schedule of the 4-week yoga program.

10.2196/80287 Multimedia Appendix 7 Schedule of the 4-week self-care program based on cognitive behavioral therapy.

10.2196/80287 Checklist 1 CONSORT-eHEALTH checklist.

## Abbreviations

CBT: cognitive behavioral therapy
CONSORT: Consolidated Standards of Reporting Trials
CSRS: Cognitive Stress Response Scale
*DSM-5*: *Diagnostic and Statistical Manual of Mental Disorders* (Fifth Edition)
DSM-IV: Diagnostic and Statistical Manual of Mental Disorders, Fourth Edition
EEG: electroencephalography
FFMQ: Five Facet Mindfulness Questionnaire
FWER: family-wise error rate
HRV: heart rate variability
*ICD-10*: *International Statistical Classification of Diseases, Tenth Revision*
K-FFMQ: Korean version of the Five Facet Mindfulness Questionnaire
K-MAIA: Korean version of the Multidimensional Assessment of Interoceptive Awareness
KOSS: Korean Occupational Stress Scale
MLM: multivariate linear model
PCL-5: Posttraumatic Stress Disorder Checklist-5
PHQ-9: Patient Health Questionnaire-9
PPG: photoplethysmograph
PSS: Perceived Stress Scale
PTSD: posttraumatic stress disorder
RCT: randomized controlled trial
