# Supplementary material for: Effectiveness of Mobile-Delivered Exercise and Yoga Programs on Depressive Symptom Reduction in Employees: Randomized Controlled Trial
Source: JMIR Hum Factors. 2026 Sep 1;13:e80287. doi: 10.2196/80287 (PMC13539178; doi:10.2196/80287)
Supplement: Supplementary file 2 [file humanfactors-v13-e80287-s001.docx]

**User-satisfaction and usability questionnaire: items and item-level results**

At their final visit, participants completed an anonymous, structured user-satisfaction and usability questionnaire about the fitness platform and the assigned exercise or yoga program. The questionnaire comprised four 4-point Likert-scale items (Q1–Q4; 1 = strongly disagree, 2 = disagree, 3 = agree, 4 = strongly agree) and six closed-ended categorical items (Q5–Q10): three single-response items (Q7, Q8, Q10) and three multiple-response items in which up to two options could be selected (Q5, Q6, Q9). Because Q1–Q4 referred specifically to the assigned exercise or yoga program, they were compared between the exercise and yoga groups using independent-samples t tests with Cohen’s d. The categorical items (Q5–Q10) were summarized as frequencies and percentages, and between-group differences in the endorsement of each response option were examined using Fisher’s exact tests. All analyses were exploratory and were not adjusted for multiple comparisons.

**A. Questionnaire items and response options**

| **Item** | **Type** | **Question and response options** |
| --- | --- | --- |
| **Q1** | Likert (4-point) | During the clinical trial, did the exercise (or yoga) program you participated in help relieve your stress? |
| **Q2** | Likert (4-point) | During the clinical trial, did the exercise (or yoga) program help you manage your mental health? |
| **Q3** | Likert (4-point) | Overall, was the platform you used during the study helpful for your health management? |
| **Q4** | Likert (4-point) | Was the platform you used during the study easy to use? |
| **Q5** | Multiple-response (up to 2) | What would you like to see improved in a platform service for physical and mental health? (1, more/varied programs; 2, performance reports; 3, sharing/communication with others; 4, stronger monitoring of completion/motivation; 5, autonomy-based platform; 6, other) |
| **Q6** | Multiple-response (up to 2) | What was the most inconvenient aspect while using the platform? (1, availability across multiple devices; 2, additional information such as subtitles/narration; 3, difficulty keeping up with the guided exercise pace; 4, improved usability of screen view/interface/speed; 5, insufficient usage guidance; 6, other) |
| **Q7** | Single-response | Have you used a health-management platform before, and do you intend to use one in the future? (1, currently using; 2, used before but no longer; 3, never used and no intention to; 4, never used but would try a good app) |
| **Q8** | Single-response | If you would use an exercise program, what is the main reason? (1, trust/expectation of physical and mental benefit; 2, newly starting exercise; 3, higher motivation than exercising alone; 4, access to varied information on the platform; 5, cost savings vs private facilities; 6, other) |
| **Q9** | Multiple-response (up to 2) | If you use or intend to use a health-management platform, which functions would you mainly use? (1, recording stress and psychological state; 2, referring to a variety of exercise programs; 3, learning exercise methods from programs; 4, posture improvement via movement feedback; 5, recommendation of a suitable program; 6, other) |
| **Q10** | Single-response | What is the greatest difficulty in using an exercise program? (1, lack of time due to work/leisure; 2, lack of adequate personal space; 3, lack of personal will to continue; 4, concurrently doing other exercise; 5, other) |

**B. Likert-scale items (Q1–Q4): comparison between the exercise and yoga groups**

| **Item** | **Exercise, mean (SD)** | **Yoga, mean (SD)** | **t** | **P value** | **Cohen's d** |
| --- | --- | --- | --- | --- | --- |
| Q1. Helpfulness for stress relief | 2.33 (0.66) | 2.83 (0.70) | −2.45 | .018 | 0.73 |
| Q2. Helpfulness for mental health management | 2.48 (0.60) | 2.88 (0.61) | −2.20 | .033 | 0.66 |
| Q3. Platform helpful for overall health management | 2.62 (0.67) | 2.96 (0.69) | −1.67 | .103 | 0.50 |
| Q4. Ease of use of the platform | 2.33 (0.66) | 2.50 (0.83) | −0.74 | .466 | 0.22 |

*Higher scores indicate greater agreement (1=strongly disagree to 4=strongly agree). P values are from independent-samples t tests and are not corrected for multiple comparisons.*

**C. Multiple-choice items (Q5–Q10): response frequencies and between-group comparisons**

**Q5. Desired improvements**

| **Response option** | **Exercise, n (%)** | **Yoga, n (%)** | **Fisher P** |
| --- | --- | --- | --- |
| 1. More / a greater variety of programs | 11 (52.4) | 8 (33.3) | .237 |
| 2. Performance reports | 6 (28.6) | 9 (37.5) | .752 |
| 3. Sharing / communication with others | 2 (9.5) | 1 (4.2) | .592 |
| 4. Stronger monitoring of completion (motivation) | 2 (9.5) | 7 (29.2) | .143 |
| 5. Autonomy-based platform | 3 (14.3) | 4 (16.7) | 1.000 |
| 6. Other | 8 (38.1) | 4 (16.7) | .176 |

**Q6. Perceived inconveniences**

| **Response option** | **Exercise, n (%)** | **Yoga, n (%)** | **Fisher P** |
| --- | --- | --- | --- |
| 1. Availability across multiple devices | 16 (76.2) | 18 (75.0) | 1.000 |
| 2. Additional information (subtitles, narration) | 2 (9.5) | 6 (25.0) | .252 |
| 3. Difficulty following the guided exercise pace | 5 (23.8) | 1 (4.2) | .083 |
| 4. Improved usability of interface / speed | 7 (33.3) | 13 (54.2) | .231 |
| 5. Insufficient usage guidance | 3 (14.3) | 0 (0.0) | .094 |
| 6. Other | 4 (19.0) | 5 (20.8) | 1.000 |

**Q7. Prior and intended platform use**

| **Response option** | **Exercise, n (%)** | **Yoga, n (%)** | **Fisher P** |
| --- | --- | --- | --- |
| 1. Currently using | 2 (9.5) | 1 (4.2) | .592 |
| 2. Used before but no longer | 8 (38.1) | 5 (20.8) | .323 |
| 3. Never used, no intention to use | 2 (9.5) | 5 (20.8) | .422 |
| 4. Never used, would try a good app | 9 (42.9) | 13 (54.2) | .554 |

**Q8. Main reason for using an exercise program**

| **Response option** | **Exercise, n (%)** | **Yoga, n (%)** | **Fisher P** |
| --- | --- | --- | --- |
| 1. Trust / expectation of benefit | 5 (23.8) | 10 (41.7) | .342 |
| 2. Newly starting exercise | 4 (19.0) | 1 (4.2) | .169 |
| 3. Higher motivation than exercising alone | 7 (33.3) | 4 (16.7) | .299 |
| 4. Access to varied platform information | 2 (9.5) | 3 (12.5) | 1.000 |
| 5. Cost savings vs private facilities | 1 (4.8) | 3 (12.5) | .611 |
| 6. Other | 2 (9.5) | 1 (4.2) | .592 |

**Q9. Preferred platform functions**

| **Response option** | **Exercise, n (%)** | **Yoga, n (%)** | **Fisher P** |
| --- | --- | --- | --- |
| 1. Recording stress / psychological state | 6 (28.6) | 9 (37.5) | .752 |
| **2. Referring to a variety of exercise programs** | **12 (57.1)** | **6 (25.0)** | **.037** |
| 3. Learning exercise methods from programs | 0 (0.0) | 3 (12.5) | .236 |
| 4. Posture improvement via movement feedback | 11 (52.4) | 10 (41.7) | .556 |
| 5. Recommendation of a suitable program | 7 (33.3) | 10 (41.7) | .759 |
| 6. Other | 1 (4.8) | 0 (0.0) | .467 |

**Q10. Greatest difficulty in using an exercise program**

| **Response option** | **Exercise, n (%)** | **Yoga, n (%)** | **Fisher P** |
| --- | --- | --- | --- |
| 1. Lack of time (work / leisure) | 5 (22.7) | 4 (16.7) | .718 |
| 2. Lack of adequate personal space | 5 (22.7) | 1 (4.2) | .090 |
| 3. Lack of personal will to continue | 10 (45.5) | 15 (62.5) | .375 |
| 4. Concurrently doing other exercise | 2 (9.1) | 3 (12.5) | 1.000 |
| 5. Other | 0 (0.0) | 1 (4.2) | 1.000 |

*Percentages are within group; multiple selections were permitted for Q5, Q6, and Q9, so column percentages may sum to more than 100%. P values are from Fisher's exact tests comparing the proportion endorsing each option between groups and are not corrected for multiple comparisons. The only statistically significant difference (shown in bold) was for Q9 option 2.*
