# Supplementary material for: Effectiveness of Mobile-Delivered Exercise and Yoga Programs on Depressive Symptom Reduction in Employees: Randomized Controlled Trial
Source: JMIR Hum Factors. 2026 Sep 1;13:e80287. doi: 10.2196/80287 (PMC13539178; doi:10.2196/80287)
Supplement: Supplementary file 4 [file humanfactors-v13-e80287-s003.docx]

**Schedule for the 4-week adaptive moderate-intensity exercise program**

1) Warm-up phase

| **Category** | **Type** | **Target** | **Rest Time** | **Heart**  **Rate** | **Step 1** | **Step 2** | **Step 3** | **Step 4** | **Step 5** | **Step 6** |
| --- | --- | --- | --- | --- | --- | --- | --- | --- | --- | --- |
| Warm-up | Pliability  Balance  Core | Dynamic  stretching | Rest time  (10 seconds)  between sets | Below 120 bpm | Kneeling ip flexible  stretch | Thoracic  rotation  stretch | 4-way  half launch | Twist knee  & toe kick | Slow pitch | Jumping jack |
|  |  |  |  |  | 1 set  (number of times) | 1 set  (number of times) | 1 set  (number of times)) | 1 set  (number of times) | 1 set  (number of times) | 1 set  (number  of times) |
|  |  |  |  |  | 5 round  trips left/right | 5 round  trips left/right | 2 round  trips left/right | 10 round  trips left/right | 10 round  trips left/right | 10 round  trips left/right |
|  |  |  |  |  | Intensity | Intensity | Intensity | Intensity | Intensity | Intensity |
|  |  |  |  |  | 3.0 | 4.0 | 4.0 | 2.0 | 5.0 | 5.0 |
|  | Participants were instructed to follow self-stretching and joint mobility exercises before and during exercise sessions.  Examples: ankle (rotation, lift, raise), knee (rotation, extension, curl), hip joint (multidirectional, kicks)  The target heart rate for dynamic stretching was set at below 120 bpm. | | | | | | | | | |

2) Training phase

| **Category** | **Type** | **Target** | **Rest Time** | **Heart**  **Rate** | **Step1** | **Step2** | **Step3** | **Step4** | **Step5** | **Step6** | **Step7** |
| --- | --- | --- | --- | --- | --- | --- | --- | --- | --- | --- | --- |
| Training | Muscular  strength | Pliability  Balance  Core | Steps 1, 2, 3,  and 4  have 30 seconds  of rest time  in between steps  Steps 5, 6, and 7  have 10 seconds  of rest time  in between steps | Below  160 bpm | Band squat  &  Shoulder  press | Spider  push-up | Push-up  &  Rotation | Side plank | <Interval  circuit >  Twist  crunch | <Interval  circuit>  Mountain  climber | <Interval  circuit>  Burpee test |
|  |  |  |  |  |  | One  round trip left/right | One  round trip left/right | Perform  in each  direction left/right | One  round trip left/right | One  round trip left/right |  |
|  |  |  |  |  | 15 times | 10 times | 10 times | (Left/  right)  10 times | Maintain maximum  speed  (10 seconds) | Maintain  maximum  speed  (10 seconds) | 10 times |
|  |  |  |  |  | Intensity | Intensity | Intensity | Intensity | Intensity | Intensity | Intensity |
|  |  |  |  |  | 6.0 | 8.0 | 7.0 | 7.0 | 8.0 | 9.0 | 10.0 |
|  | The training phase was performed in the following sequence:  step 1→step 2→step 3→step 4→step 5→step 6→step 7→step 5→step 6→step 7  The target heart rate range during the training phase was set at below 160 bpm. | | | | | | | | | | |

3) Cool-down phase

| **Category** | **Type** | **Rest Time** | **Heart**  **Rate** | **Step 1** | **Step 2** | **Step 3** | **Step 4** | **Step 5** | **Step 6** |
| --- | --- | --- | --- | --- | --- | --- | --- | --- | --- |
| Cool-down | Balance stabilization  Static  stretching  Mindset & relaxation movements | 10 seconds  of rest  between  sets | Below  90 bpm | Chest  stretching | Shoulder turn | Cardiac muscle stretching | Grab your toes  with one hand  and pull them back | Calf stretch | Cow &  Cat  stretch |
|  |  |  |  | Number of times | Number of times | Number of times | Number of times | Number of times | Number of times |
|  |  |  |  | Instructions:  Stand up straight  and  take a deep breath.  When you inhale,  spread your arms  to open your chest.  (1 round trip with  arms outstretched  and together) | Perform  clockwise  /counter-  clockwise | Perform  left/right  direction | Perform left/right direction | Stretch your calf by spreading your feet  forward and back,  placing your back  heel on the floor,  and bending the knee  of your front leg. | - |
|  |  |  |  | 10 round trips | Clockwise  10 times  Counter-  clockwise  10 times | Left stretch  5 seconds  Right stretch  5 seconds | Left stretch  5 seconds  Right stretch  5 seconds | Left stretch  5 seconds  Right stretch  5 seconds | 5 times |
|  |  |  |  | Intensity | Intensity | Intensity | Intensity | Intensity | Intensity |
|  |  |  |  | 2.0 | 2.0 | 2.0 | 2.0 | 2.0 | - |
|  |  |  |  | Standing movement | Sitting movement | Sitting movement | Meditation |  |  |
|  | The cool-down phase included balance training movements,  static stretching targeted stabilization of the major muscle groups and progressively reduction of exercise intensity.  The target heart rate for the cool-down phase was set at below 90 bpm. | | | | | | | | |
