# Supplementary material for: Effectiveness of Mobile-Delivered Exercise and Yoga Programs on Depressive Symptom Reduction in Employees: Randomized Controlled Trial
Source: JMIR Hum Factors. 2026 Sep 1;13:e80287. doi: 10.2196/80287 (PMC13539178; doi:10.2196/80287)
Supplement: Supplementary file 5 [file humanfactors-v13-e80287-s004.docx]

**Examples of movements included in the exercise program protocol**

1) Warm-up phase

| **Category** | **Type** | **Movement 1** | **Movement 2** | **Movement 3** | **Movement 4** |
| --- | --- | --- | --- | --- | --- |
| Warm-up | Tensor Fasciae  Latae  stretching |  |  |  |  |
|  |  | Step one foot forward and  the other back  Raise your arms overhead | Extend your arm to the  side | Repeat on the opposite side | Perform  in the opposite direction |
|  | Main parts of use | Tensor Fasciae Latae muscle | | | |
|  | Standing Adductor stretching |  | |  | |
|  |  | Place your hands on your waist and stretch your legs  side to side | | Perform in the opposite direction | |
|  | Main parts of use | Adductor muscles, hip joints, etc. | | | |

2) Training phase

| Training Phase | Hip bridge |  |  |
| --- | --- | --- | --- |
|  |  | Lie on the floor with your knees bent. Position your feet about hip-width apart | After creating a spinal energy state, slowly lift your hips. Add further stimulation to the gluteus by stopping the movement  for a while with the hips raised. |
|  | Main parts of use | Enhance lumbar and pelvic stability and strengthen gluteal and hip movements.  Rear deltoid muscle, gluteus muscle, hamstring, femoral muscles. | |
|  | Additional instructions | Movements with excessive waist lifting or improper spinal alignment can strain unnecessary muscles or lead to injury. | |
|  | Seated knee-up |  |  |
|  |  | Sit on the floor with both hands placed behind your hips. Lean your body back about 30 degrees. | Perform the movement by bending your back and knees slightly, bringing your chest toward your knees |
|  | Main parts of use | [Musculus rectus abdominis](https://en.dict.naver.com/#/entry/enko/e4e49e8fa58d4f3d960068695923ada4), [oblique abdominal](https://en.dict.naver.com/#/entry/enko/7ff5ed12a5744e7687ad75c466713512), [femoral muscles](https://en.dict.naver.com/#/entry/enko/a692e991aed34b6787103cf744f7c328) | |
|  | Additional instructions | Keep your center of gravity toward the back to maintain your balance and prevent losing control of your body. | |
