# Supplementary material for: Effectiveness of Mobile-Delivered Exercise and Yoga Programs on Depressive Symptom Reduction in Employees: Randomized Controlled Trial
Source: JMIR Hum Factors. 2026 Sep 1;13:e80287. doi: 10.2196/80287 (PMC13539178; doi:10.2196/80287)
Supplement: Supplementary file 6 [file humanfactors-v13-e80287-s005.docx]

**Example of movements in the yoga program**

| 2.5 | Head-to-knee pose  (Janu Sirsasana) |  | 1. Sit on the ground with your legs stretched out in front of you  2. Stretch your right leg forward, bend your left knee, placing the left heel near your perineum, and press the sole of your left foot against the inner  side of your right thigh.  3. Create a 90-degree angle between your legs  4. Bend forward so that your head touches your right shin, between your chest and shoulders on both sides.  5. When the stretch in your left waist become comfortable, align both shoulders horizontally  6. Lengthen your spine as if the crown of your head is reaching forward  7. Repeat on the opposite side |
| --- | --- | --- | --- |
| 2.6 | Seated forward bend  (Paschimottanasana) |  | 1. Sit on the ground with your legs stretched out in front of you  2. Hold your toes or soles with both hands and stretch your spine  3. Breathe out and stretch your spine toe lower our upper body  4. Breathe out and bring the top of your head and the top of your foot close by using your elbow |
| 2.7 | Child’s pose (Balasana) |  | 1. Close your eyes and concentrate on your inner self  2. Feel breathing and relax your spine  3. Relax your arms and shoulders |
