# Supplementary material for: Effectiveness of Mobile-Delivered Exercise and Yoga Programs on Depressive Symptom Reduction in Employees: Randomized Controlled Trial
Source: JMIR Hum Factors. 2026 Sep 1;13:e80287. doi: 10.2196/80287 (PMC13539178; doi:10.2196/80287)
Supplement: Supplementary file 7 [file humanfactors-v13-e80287-s006.docx]

**Schedule of the 4-week yoga program**

1) Warm-up phase

| **No** | **Practices** | **Duration**  **(minutes)** | **Repetitions** |
| --- | --- | --- | --- |
| 1.1 | Classic seated pose (Siddhasana) | 2 | 1 |
| 1.2 | Neck rolls | 4 | 1 |
| 1.3 | Shoulder rolls & arm stretches | 2 | 1 |
| 1.4 | Ankle stretch | 2 | 1 |
| 1.5 | Thigh stretch | 2 | 1 |
| 1.6 | Stick pose (Yastikasana) | 1 | 1 |
| 1.7 | Knee stretch | 1 | 1 |

2) Training phase

| **No** | **Practices** | **Duration**  **(minutes)** | **Repetitions** |
| --- | --- | --- | --- |
| 2.1 | Backward roll | 2 | 2 |
| 2.2 | Cow face pose (Gomukhasana) | 3.5 | 1 |
| 2.3 | Pigeon pose  (Eka Pada Rajakapotasana) | 2 | 3 |
| 2.4 | Butterfly pose (Baddha Konasana) | 4 | 3 |
| 2.5 | Head-to-knee pose (Janu Sirsasana) | 4 | 3 |
| 2.6 | Seated forward bend (Paschimottanasana) | 2 | 1 |
| 2.7 | Child’s pose (Balasana) | 1 | 1 |
| 2.8 | Bridge pose  (Setu bandha Sarvangasana) | 4.5 | 5 |
| 2.9 | Cat & Dog Stretch | 2 | 5 |
| 2.10 | Downward dog pose (Adho mukha svanasana)  & Warrior pose (Virabhadrasana) | 7 | 1 |

3) Cool-down phase

| **No** | **Practices** | **Duration**  **(minutes)** | **Repetitions** |
| --- | --- | --- | --- |
| 3.1 | Happy baby pose | 1 | 1 |
| 3.2 | Relaxation on supine pose | 3 | - |
