# Supplementary material for: Effectiveness of Mobile-Delivered Exercise and Yoga Programs on Depressive Symptom Reduction in Employees: Randomized Controlled Trial
Source: JMIR Hum Factors. 2026 Sep 1;13:e80287. doi: 10.2196/80287 (PMC13539178; doi:10.2196/80287)
Supplement: Supplementary file 8 [file humanfactors-v13-e80287-s007.docx]

**Schedule of the 4-week self-care program based on cognitive behavioral therapy**

| Week 1 Understanding Stress | Day 1: Stress Response  Day 2: Recognizing Stress  Day 3: Identifying Stress Warning Signs |
| --- | --- |
| Week 2  The Seeds of Stress | Day 4: Desires that Create Stress  Day 5: Desires and Thoughts that Create Stress  Day 6: Thoughts that Create Stress |
| Week 3  Stress Management | Day 7: Support Systems  Day 8: Resilience  Day 9: Mindfulness |
| Week 4  Stress Management | Day 10: Sleep Hygiene  Day 11: Performance Does Not Define Your Worth  Day 12: List of Enjoyable Activities |
